# Integrated bioinformatic analysis and experimental validation for exploring the key molecular of brain inflammaging

**DOI:** 10.3389/fimmu.2023.1213351

**Published:** 2023-07-10

**Authors:** Zhixin Du, Yaohui Wang, Liping Yang, Tong Zhang, Yu Jiang, Zhenqiang Zhang

**Affiliations:** ^1^ School of Medicine, Henan University of Chinese Medicine, Zhengzhou, China; ^2^ Academy of Chinese Medical Sciences, Henan University of Chinese Medicine, Zhengzhou, China

**Keywords:** inflammaging, single cell, CX3CL1, immunosenescence, macrophage

## Abstract

**Aims:**

Integrating bioinformatics and experimental validation to explore the mechanisms of inflammaging in the Brain.

**Method:**

After dividing the GSE11882 dataset into aged and young groups, we identified co-expressed differentially expressed genes (DEGs) in different brain regions. Enrichment analysis revealed that the co-expressed DEGs were mainly associated with inflammatory responses. Subsequently, we identified 12 DEGs that were related to the inflammatory response and used the DGIdb website for drug prediction. By using both the least absolute shrinkage and selection operator (LASSO) and random forest (RF), four biomarkers were screened and an artificial neural network (ANN) was developed for diagnosis. Subsequently, the biomarkers were validated through animal studies. Then we utilized AgeAnno to investigate the roles of biomarkers at the single cell level. Next, a consensus clustering approach was used to classify the aging samples and perform differential analysis to identify inflammatory response-related genes. After conducting a weighted gene co-expression network analysis (WGCNA), we identified the genes that are correlated with both four brain regions and aging. Wayne diagrams were used to identify seven inflammaging-related genes in different brain regions. Finally, we performed immuno-infiltration analysis and identified macrophage module genes.

**Key findings:**

Inflammaging may be a major mechanism of brain aging, and the regulation of macrophages by CX3CL1 may play a role in the development of inflammaging.

**Significance:**

In summary, targeting CX3CL1 can potentially delay inflammaging and immunosenescence in the brain.

## Graphical Abstract

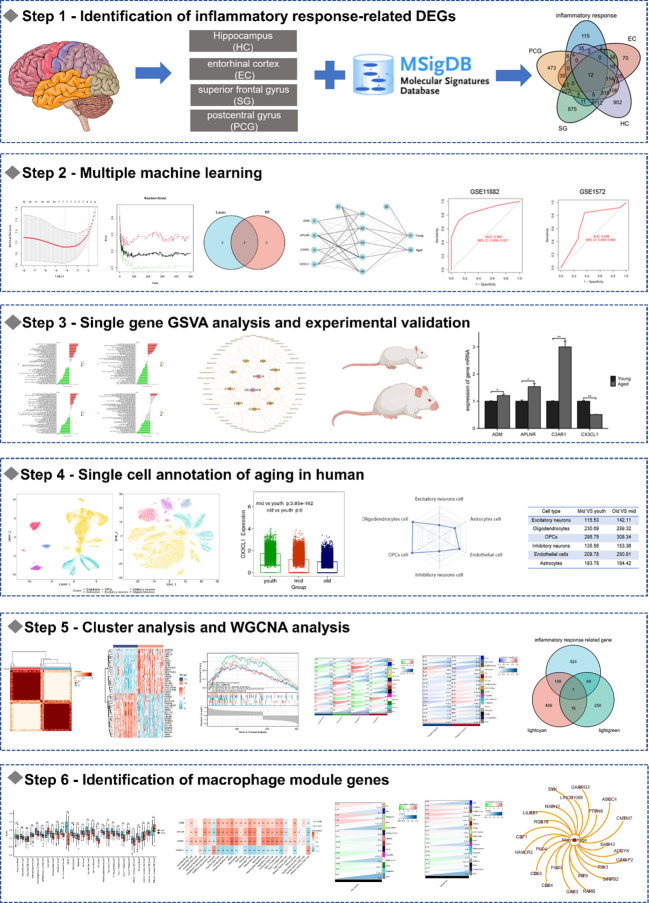

## 1 Introduction

Aging refers to the gradual loss of physiological integrity and functional decline in biological processes, leading to impaired function and increased risk of death, often caused by dysregulated mitochondrial metabolism, cellular function, tissue failure, and overall body function (1, 2). In the adult brain, a balance between pro- and anti-inflammatory factors must be maintained. However, as we age, this balance tends to tip towards pro-inflammatory cytokines (3). This imbalance makes the aging brain more susceptible to stress and disease. And the low level of chronic inflammation has become the sign and potential driving factor of brain aging (4, 5). Typically, older brains have higher levels of inflammatory markers in their cells and tissues, resulting in ongoing changes in cellular senescence (6). The immune system and immune cells gradually age, resulting in disrupted activation and elimination of inflammatory responses (7). Macrophages are a crucial component of the innate immune system, and their “polarization” phenomenon, which allows for a dynamic shift in phenotype and function depending on the specific environment, is critical for regulating inflammation development (8, 9). Inflammation, immune system and aging are closely related and are one of the important directions of anti-aging research.

Numerous studies indicate that aging of both the body and brain can be delayed, and removing senescent cells from the brain can significantly slow down brain aging (10, 11). Bioinformatics analysis has rapidly advanced and become a valuable tool for identifying disease-related genes (12). In this study, we focused on brain aging in different regions and aimed to identify potential biomarkers and molecular mechanisms involved. By analyzing the expression profile of GSE11882 from the gene expression omnibus (GEO) database, we found that inflammaging plays a crucial role in brain aging. By using the DGIdb website, we predicted drug targets for 12 inflammatory response-related DEGs and developed an artificial neural network (ANN) based on four biomarkers selected by both LASSO and RF. The biomarkers were validated through animal studies, and their roles in single cells were investigated using AgeAnno. Subsequently, we performed consensus clustering and weighted gene co-expression network analysis (WGCNA) to further investigate the mechanisms of inflammaging of the brain, with the ultimate goal of providing insights to aid in slowing down brain aging treatments.

## 2 Materials and methods

### 2.1 Data collection and processing

GSE11882 and GSE1572 dataset were obtained from the in the GEO database (http://www.ncbi.nlm.nih.gov/geo). GSE11882 dataset was used as the date set, comprising of 173 postmortem brain tissue samples obtained from four distinct regions of the brain: 43 from the hippocampus (HC), 39 from the entorhinal cortex (EC), 48 from the superior frontal gyrus (SG), and 43 from the postcentral gyrus (PCG) (12). We divided the sample into the young group (age range: 20-59 years) and the aged group (age range: 60-99 years). And GSE1572 dataset was used as the validation set, included 22 aged samples and 24 young samples.

### 2.2 Identification of inflammatory response-related differentially expressed genes and functional enrichment analysis

The “limma” package was utilized to identify differentially expressed genes (DEGs), which were selected based on a threshold of |log2FC|>0.5 (where FC denotes fold change) and *P*<0.05. Enrichment analysis of gene ontology (GO) and kyoto encyclopedia of genes and genomes (KEGG) pathways was performed with the R package “clusterProfiler”. The cutoff values for q-value and p-value were set to 0.05.

The results of the enrichment analysis were visualized using R software. Following that, inflammatory response related - DEGs were identified through their intersection in the DEGs and 200 inflammatory response related genes from MsigDB (http://www.broad.mit.edu/gsea/msigdb/). Then, GO terms and KEGG pathway were also performed using the R cluster profiler package.

### 2.3 Screening for potential compounds

Drug gene interaction database (DGIdb) (https://www.dgidb.org/) provides information about drug and gene interactions. In this study, the DGIdb be used to identify potential small-molecule substances targeting inflammatory response-related DEGs.

### 2.4 Machine learning

We used two machine learning algorithms to filter genes, including support vector machine recursive feature elimination (SVM-RFE) and least absolute shrinkage and selection operator (LASSO). LASSO is a regression analysis method that combines feature selection and regularization simultaneously. And SVM-RFE employ the inductive principles of structural risk minimization. The genes screened by LASSO and SVM-RFE were used for further analysis.

### 2.5 Artificial neural network construction

Using the “neuralnet” and “neuralnettools” packages in R software, a model based on artificial neural network (ANN) was constructed for the biomarkers. The model had five hidden nodes. A classification model was created for aged utilizing the “gene score” information. The ANN’s predictive performance was assessed by analyzing the receiver operating characteristic (ROC) curves on both the GSE11882 and GSE1572 datasets.

### 2.6 GSVA analysis

According to the median expression levels of biomarkers in the GSE11882 dataset, the aged samples were divided into high and low-expression group. The study employed single-gene set variation analysis (GSVA) to clarify the enriched KEGG pathways, with the gene set “c2.cp.kegg. v7.4. symbols. gmt” serving as the reference. Gene sets with *P* < 0.05 were considered to be significantly enriched.

### 2.7 Animal experiments

Male Wister rats were assigned to two groups—the young group (3 months; n=6) and the aged group (24 months; n=6), and housed under constant environmental conditions (a temperature of 22 ± 2°C and a humidity of 50–70%) on a light/dark cycle of 12 h. All animal experiments were approved by the experimental animals’ welfare and ethical inspection of beijing vital river laboratory animal technology Co., Ltd. The procedures were approved by the Animal Ethics Committee of Henan University of Chinese Medicine (permit number: DWLL202108003).

### 2.8 Molecular validation

To measure the expression of ADM, APLNR, C3AR1 and CX3CL1, we used Real time-quantitative PCR (RT-PCR). Total RNA was extracted from hippocampal tissues using Trizol reagent (Servicebio, G3013). The mRNA was reverse transcribed to cDNA using the Servicebio^®^RT First Strand cDNA Synthesis Kit (Servicebio, G3337), followed by qPCR using the SYBR Green qPCR Master Mix (None ROX) (Servicebio, G3320). We used the 2^–ΔΔCt^ method to determine the relative changes in mRNA levels among the groups. Thermal cycling conditions for RT-PCR were 95°C for 30 s, followed by 40 cycles of 95°C for 15 s and 60°C for 30 s, and finally 60°C for 60 s to 95°C for 15 s. We used GAPDH as the housekeeping gene to normalize the expressions of the target genes. The following primers were used for amplification in **Table 1**.

**Table 1:** The primer sets for associated mRNA.

Rat	Forward Primer (5′-3′)	Reverse Prime (5′-3′)	Fragment length (bp)
ADM	CTGGTTTCCATCGCCCTGAT	GTAGCTGCTGGACGCTTGTA	150
APLNR	CCTACCGGGAGTTTGACTGG	GCAGCCTTAGTCGAGCGTTA	162
C3AR1	ACCAAGAAAGCGCCTTGAGA	AACTGGTAGAGTGCGTGAGC	192
CX3CL1	GGCCGCGTTCTTTCATCTGT	GGATTGGCGAGGTCATCTTGT	95
GAPDH	CTGGAGAAACCTGCCAAGTATG	GGTGGAAGAATGGGAGTTGCT	138

### 2.9 Single-cell RNA sequencing analysis

AgeAnno (https://relab.xidian.edu.cn/AgeAnno/#/) is a powerful tool that leverages scRNA data to comprehensively characterize aging-associated genes across various human tissue cell types (13). AgeAnno allowing us to visualize cells from different age groups and identify the specific cell types that are involved in the aging process. We utilized AgeAnno to investigate the roles of biomarkers.

### 2.10 Consensus clustering

We performed unsupervised clustering analysis on the aged samples using the “ConsensusClusterPlus” software package based on the expression profiles of 12 inflammatory response related - DEGs. As a result, we classified the aged samples into two distinct clusters. We set the parameters as follows: the maximum number of clusters is 10 and the distance metric used was Pearson. The empirical cumulative distribution function plot was utilized to determine the optimal number of clusters. To determine the significance of the clusters, principal component analysis (PCA) was used. The DEGs between the two clusters were analyzed using the “limma” package, with thresholds of |log2FC|≥ 1 and *P* < 0.05.

### 2.11 GSEA analysis

We conducted enrichment analyses of GO terms and KEGG pathways using gene set enrichment analysis (GSEA), which was carried out with the R package and the MSigDB database. The cutoff criteria were set at False discovery rate (FDR) < 0.25 and *P* < 0.05.

### 2.12 WGCNA analysis

We constructed a co-expression network using the WGCNA package in order to identify the hub module that is most relevant to the aged. We used the R software package “WGCNA” to analyze gene correlations. Genes with variances exceeding 50% were selected as inputs for the analysis. To ensure that the resulting network followed a scale-free topology, we applied a soft threshold. We used hierarchical clustering and dynamic tree cutting functions to identify modules. To determine the significance of module eigengenes (MEs), we calculated their p-value and Pearson’s correlation coefficient with the disease trait. This helped us to identify the most relevant module for aging. Subsequently, we constructed a co-expression network for aged samples using the WGCNA package to identify the module most relevant to the four brain regions. We used a Venn diagram to identify genes that were associated with both aged and the brain regions.

### 2.13 Identification of macrophage-related module genes

Using the single-sample gene set enrichment analysis (ssGSEA) method from the R package, we compared the immune landscape of aged and young samples. A violin plot was generated using the “vioplot” package to visualize the 28 immune cell between 2 groups. To examine the correlations between immune cells and biomarkers, we performed Spearman’s correlation analysis. We also generated the module-macrophage correlation heat map based on the results of WGCNA analysis and immune infiltration. We then identified the macrophage-related module genes.

## 3 Result

### 3.1 Identification of DEGs

**Figure 1:**
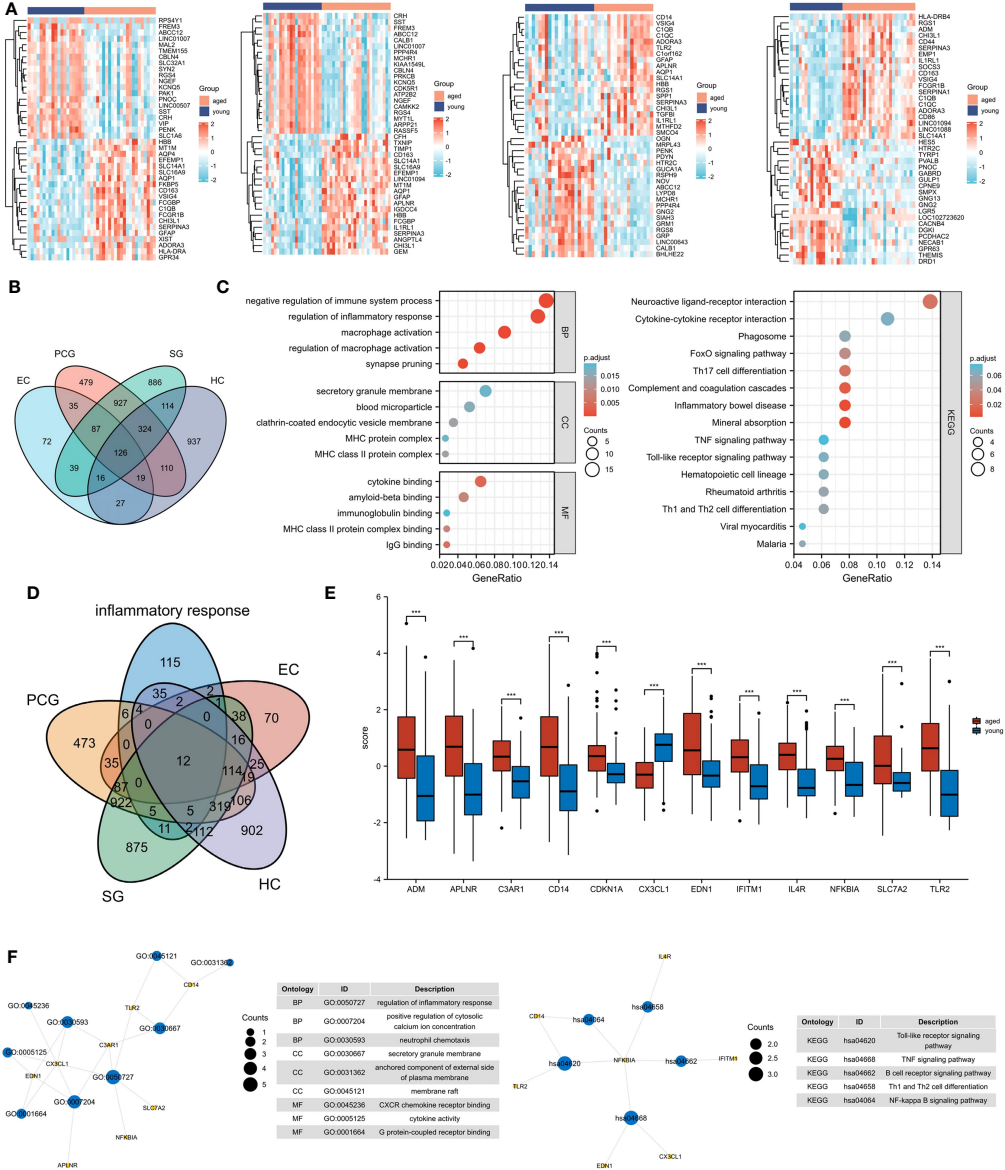
Identification of inflammatory response-related DEGs. **(A)** Heat maps of DEGs between aged and young groups in HC, SG, EC and PCG. **(B)** Venn diagrams of DEGs. **(C)** GO and KEGG pathways enriched by the DEGs. **(D)** Venn diagrams of inflammatory response-related DEGs. **(E)** Box plot of 12 inflammatory response-related DEGs , ***P <0.001. **(F)** GO and KEGG pathways enriched by the inflammatory response-related DEGs. HC, hippocampus; PCG; postcentral gyrus; SG, superior frontal gyrus; EC, entorhinal cortex.

We created a corresponding heat map (**Figure 1A**) and found 126 DEGs in four brain regions (**Figure 1B**). Our subsequent functional enrichment analysis revealed that GO terms related to cytokine production, immune system regulation, regulation of inflammatory response, macrophage activation, and cytokine binding were significantly enriched. KEGG pathway analysis identified cytokine-cytokine receptor interaction, FoxO signaling pathway, TNF signaling pathway, and phagosome as the most enriched pathways. These results indicate a strong correlation between aging and inflammatory response (**Figure 1C**).

### 3.2 Identification of inflammatory response - related DEGs

A total of 12 inflammatory response - related DEGs were identified (**Figures 1D, E**), and GO analysis indicated that the biological processes were focused on the regulation of inflammatory response, positive regulation of cytosolic and neutrophil chemotaxis. The cellular composition was focused on secretory granule membrane, anchored component of external side of plasma membrane and membrane raft. The molecular functions were mainly focused on CXCR chemokine receptor binding, cytokine activity and G protein-coupled receptor binding. And KEGG analysis showed that Toll-like receptor signaling pathway, TNF signaling pathway and B cell receptor signaling pathway were significantly enriched (**Figure 1F**).

### 3.3 Identification of the potential drugs

**Figure 2:**
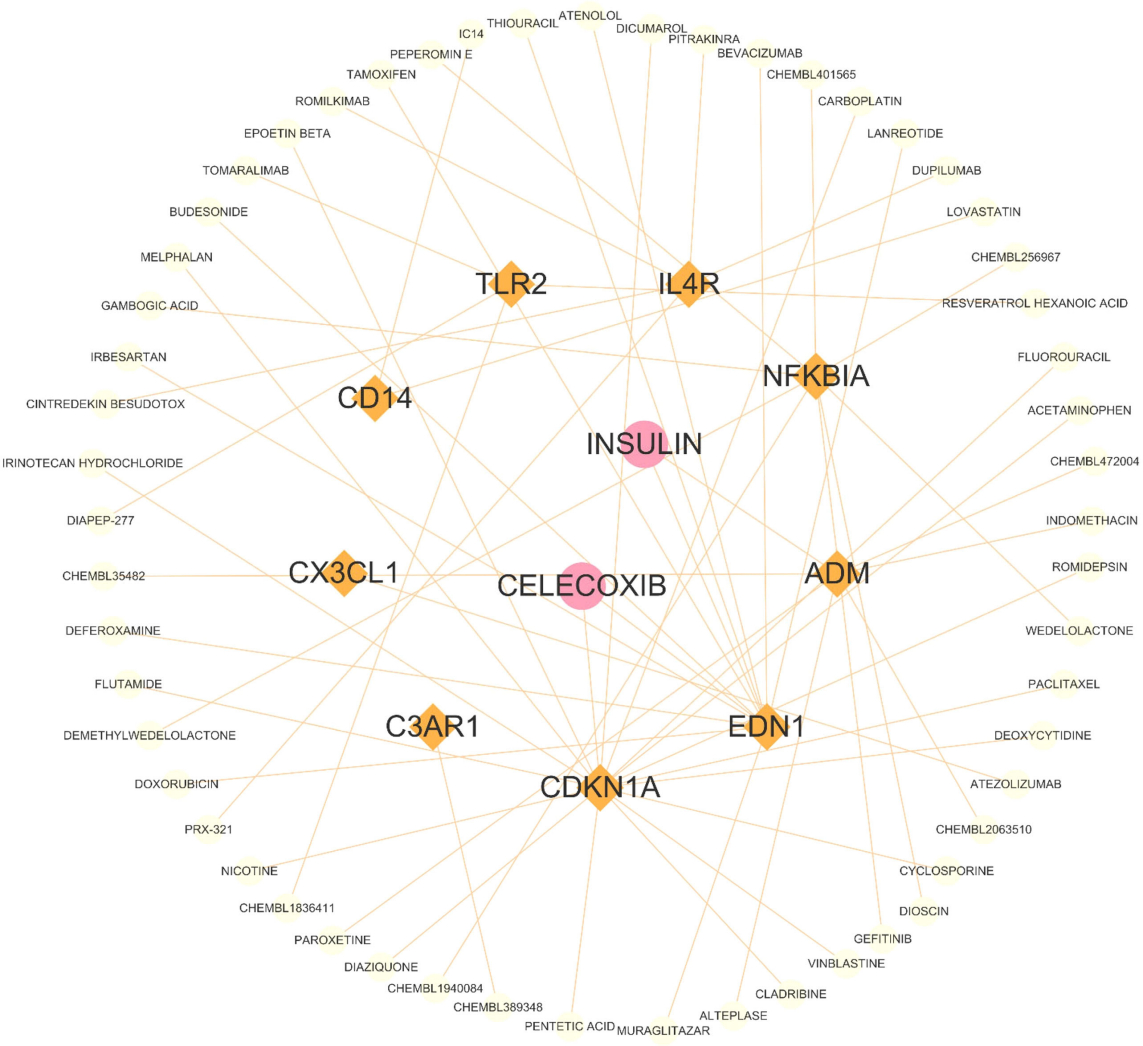
The drug and genes interaction network. Circles represent drug, and lozenges represent inflammatory response-related DEGs.

In **Figure 2**, A total of 59 gene-related compounds or medications were discovered. Notably, celecoxib and insulin can target multiple genes at the same time, indicating their potential efficacy as drugs in the future.

### 3.4 Identification of biomarkers

**Figure 3:**
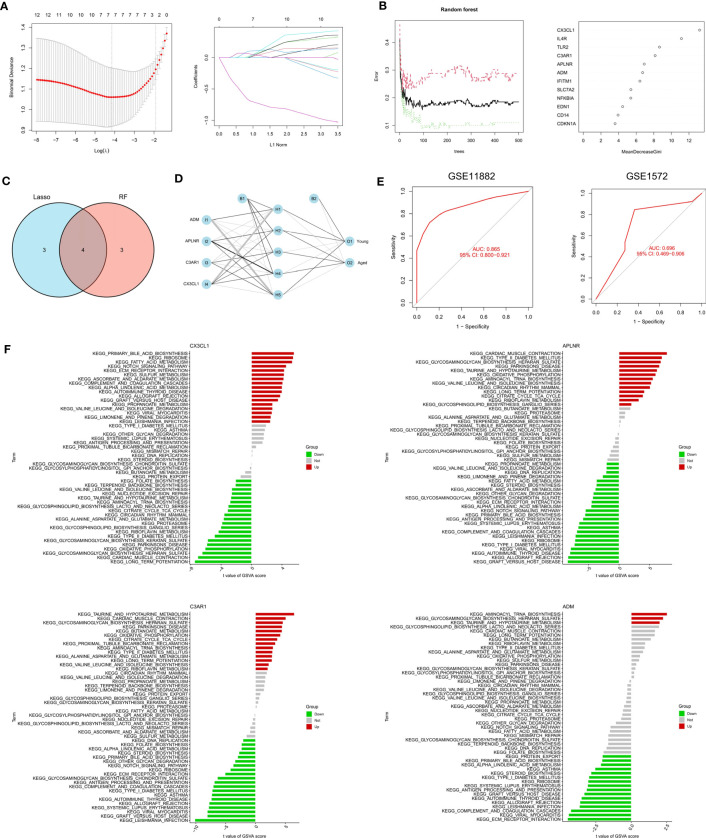
Identification and validation of the biomarkers. **(A)** RF algorithm analysis. **(B)** LASSO regression analysis. **(C)** Venn plot exhibiting the biomarkers. **(D)** The visualization of the ANN diagnostic model. **(E)** The assessment result of the GSE11882 and the testification result of the GSE1572. **(F)** GSVA analysis of CX3CL1, APLNR, C3AR1 and ADM.

The RF algorithm identified seven diagnostic genes (**Figure 3A**), while the LASSO regression algorithm identified seven potential diagnostic biomarkers (**Figure 3B**). A Venn diagram was used to identify the four genes (ADM, APLNR, C3AR1, and CX3CL1) that overlapped and were deemed robust diagnostic biomarkers (**Figure 3C**). Then, an ANN diagnostic model was developed based on gene weight (**Figure 3D**). When tested on the dataset (GSE11882), the model achieved an AUC of 0.865, while for the validation set (GSE1572) the AUC was 0.696 (**Figure 3E**), indicating high performance in diagnosing aged individuals. These results demonstrate the successful development of a robust diagnostic model capable of distinguishing between aged and young samples.

### 3.5 Single-gene GSVA analysis

After that, we performed a single-gene GSVA analysis (**Figure 3F**). The results showed that the high expression of CX3CL1 was associated with the enrichment of the notch signaling pathway, while the low expression of CX3CL1 was linked to Parkinson’s disease. The low expression of APLNR was associated with ECM receptor interaction, whereas the high expression of APLNR was associated with Parkinson’s disease. Moreover, the high expression of C3AR1 was found to be highly enriched in Parkinson’s disease, while the low expression of C3AR1 was associated with the notch signaling pathway. Finally, the high expression of ADM was mainly enriched in aminoacyl tRNA biosynthesis, while the low expression of ADM was associated with ECM receptor interaction.

### 3.6 Experimental validation

**Figure 4:**
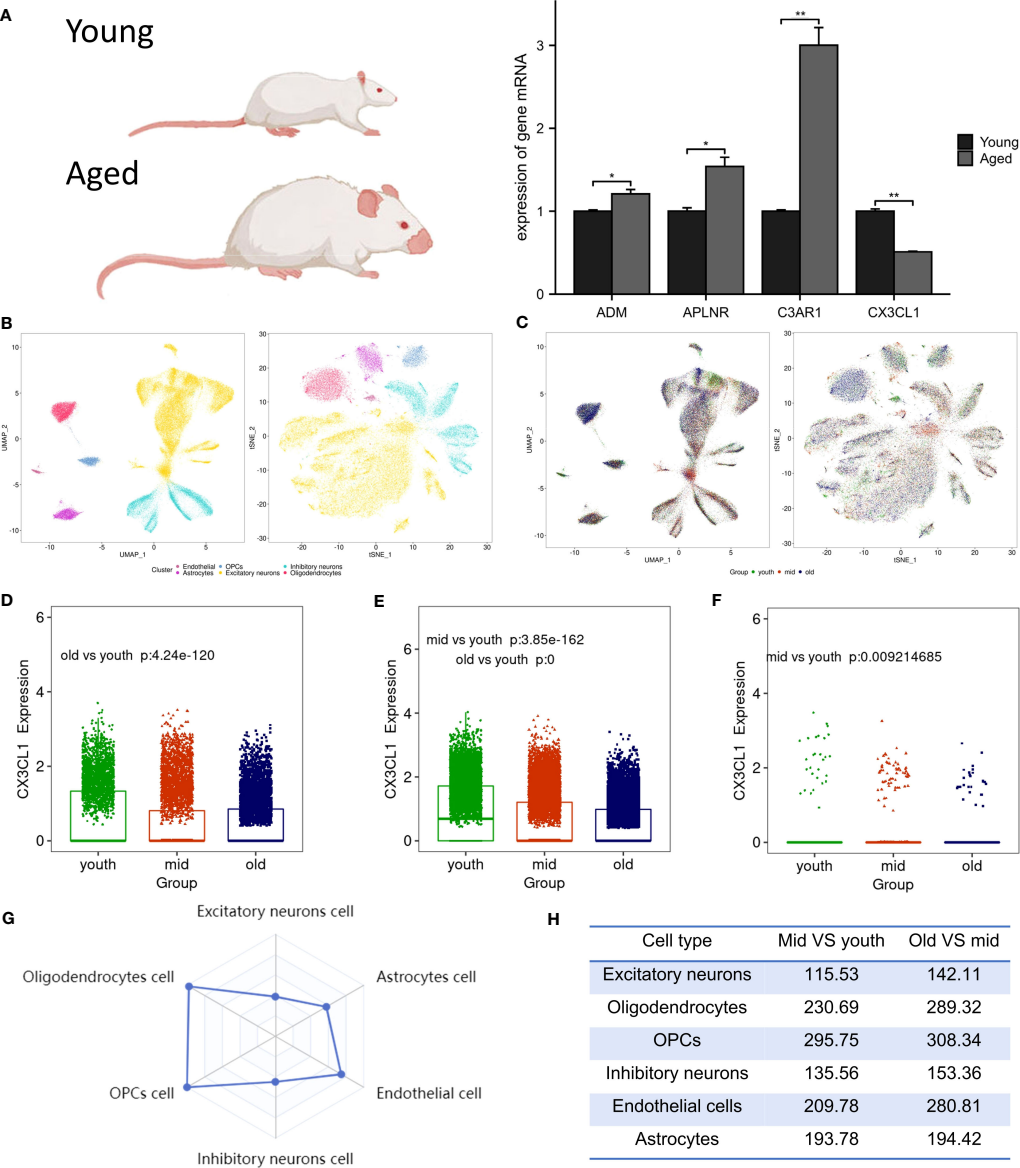
Animal experiment validation and single cell analysis. **(A)** The expression of ADM, APLNR, C3AR1 and CX3CL1 in hippocampal tissue of aged and young rat , *P <0.05 , **P <0.01. **(B)** The tSNE and UMAP plots of different cell clusters in the brain. **(C)** The tSNE and UMAP plots of cells of different age groups in the brain. **(D)** CX3CL1 expression is downregulated in inhibitory neurons after brain aging. **(E)** CX3CL1 expression is downregulated in excitatory neurons after brain aging. **(F)** CX3CL1 expression is downregulated in astrocytes after brain aging. **(G)** Radar plot of variation coefficient of CX3CL1 in six cell type. **(H)** Comparison of CX3CL1 variation coefficient.

Following RT-PCR analysis, it was discovered that the aged group exhibited significantly lower expression of CX3CL1 mRNA compared to the young group, as demonstrated in **Figure 4A**. In addition, the expression levels of ADM, C3AR1, and APLNR mRNA in the aged group were higher, in line with the findings from GSE11882.

### 3.7 Single-cell expression of biomarkers

Biomarkers were employed to differentiate six distinct cell types in brain tissue, which include excitatory neurons, inhibitory neurons, oligodendrocytes, endothelial cells, oligodendrocyte progenitor cells (OPCs), and astrocytes (**Figure 4B**). It was observed that in the aged group, the population of excitatory neurons, oligodendrocytes, and OPCs were increased, while the numbers of endothelial cells and astrocytes were decreased (**Figure 4C**). Research revealed a significant decrease in CX3CL1 expression across three cell types: inhibitory neurons (**Figure 4D**), excitatory neurons (**Figure 4E**), and astrocytes (**Figure 4F**). Notably, CX3CL1 expression was found to be most significantly reduced in excitatory neurons. Transcriptional noise refers to the molecular fluctuations that cause variations in gene expression among cells in a population. **Figure 4G** presents a radar plot that illustrates the coefficient of variation of CX3CL1 in six brain cell types, along with a table that shows how the coefficient of variation of CX3CL1 increases with aging in all cell types (**Figure 4H**). This finding suggests that dysregulation of transcriptional regulation may underlie age-related changes in CX3CL1 gene expression.

### 3.8 Cluster analysis

**Figure 5:**
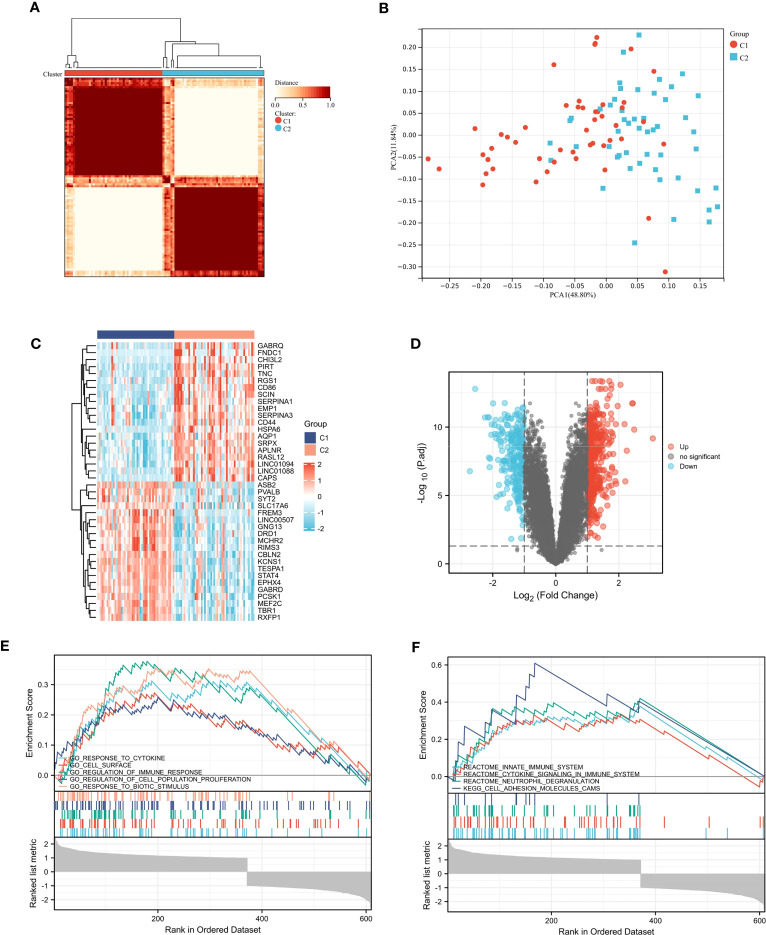
Consensus clustering analysis. **(A)** Defined two clusters and the consensus matrix heat map of their related regions. **(B)** PCA analysis showed transcriptome differences between the two subtypes. **(C, D)** The heat map and volcano map between cluster C1 and cluster C2. **(E, F)** Five representative enriched GO gene sets and KEGG pathways from GSEA.

In GSE11882, we conducted cluster analysis to categorize the aged samples into distinct molecular subtypes (**Figure 5A**). To explore the subtypes further, we performed PCA based on the expression levels of the 12 inflammatory response - related DEGs (**Figure 5B**). The resulting analysis revealed two clusters, which we named Clusters C1 and C2.

### 3.9 GSEA analysis

We identified DEGs between cluster C1 and cluster C2. The volcanic and thermal diagrams are shown in **Figures 5C, D**. Then we ran GSEA to identify molecular activities that are different between the two clusters. As shown in **Figure 5E**, GO terms were mainly enriched in response to cytokine, regulation of immune response and cell surface. And the KEGG terms were mainly enriched in innate immune system, cytokine signaling in immune system and neutrophil degranulation (**Figure 5F**).

### 3.10 WGCNA analysis

**Figure 6:**
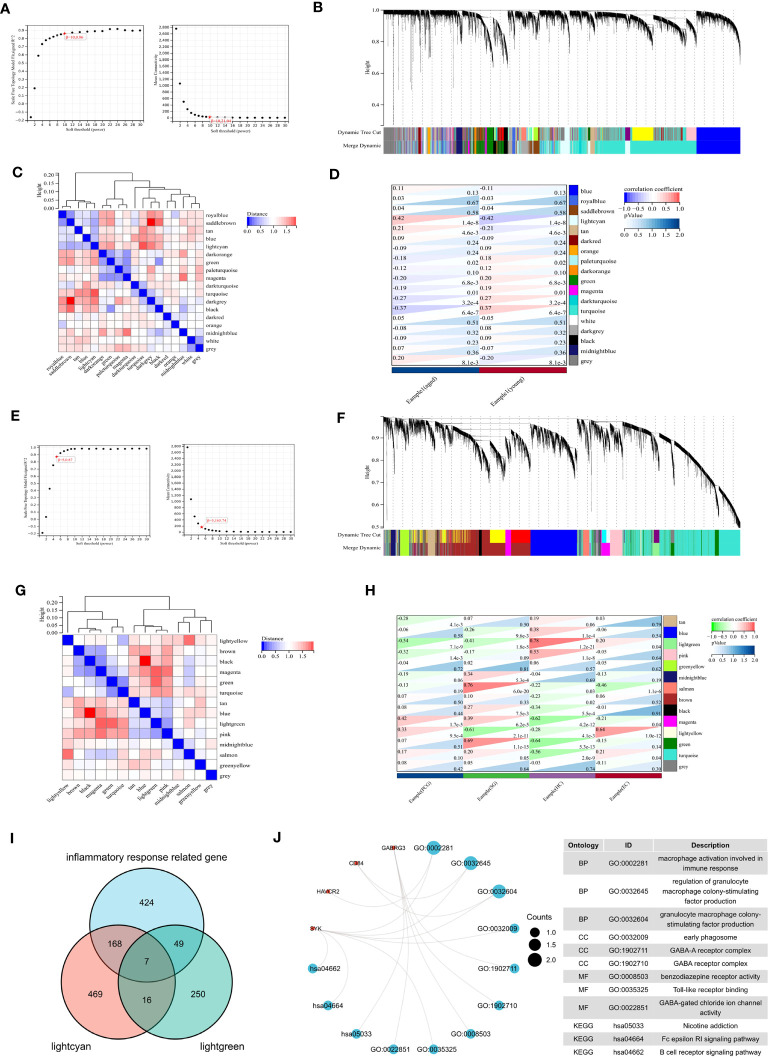
WGCNA analysis. **(A)** β =10 is selected as the soft threshold in the combined analysis of scale independence and average connectivity. **(B)** Gene co-expression modules represented by different colors under the gene tree. **(C)** Correlation heat map of modules. **(D)** Heat map of the association between modules and phenotypes. Numbers at the top and bottom brackets represent the correlation coefficient and p-value, respectively. **(E)** β =5 is selected as the soft threshold. **(F)** Gene co-expression modules represented by different colors under the gene tree. **(G)** Correlation heat map of modules. **(H)** Heat map of the association between modules and brain regions. **(I)** Venn plot exhibiting the seven inflammaging-related genes in different brain regions. **(J)** GO and KEGG pathways enriched by the inflammaging genes in different brain regions.

We conducted WGCNA analysis to identify gene modules associated with specific features. Using a soft threshold of β = 10, we constructed a scale-free network (**Figure 6A**) and merged similar modules to generate a dynamic cut tree (**Figure 6B**). And 18 modules have been generated, which can be seen in **Figure 6C** in different colors. Among the 18 modules, we found that the midnight lightcyan module was closely related to aged features (**Figure 6D**). Similarly, we performed WGCNA analysis on the aged sample using β = 5, we constructed a scale-free network (**Figure 6E**) and merged similar modules to generate a dynamic cut tree (**Figure 6F**). We obtained 14 modules, which are depicted in **Figure 6G** with distinct colors. We discovered that the lightgreen module was significantly correlated with four brain regions and most closely associated with the hippocampus (**Figure 6H**).

We then analyzed the intersection of the lightcyan module genes, lightgreen module genes, and inflammatory response related genes to identify seven inflammaging-related genes in different brain regions (**Figure 6I**). We then performed a functional enrichment analysis and the results showed that GO terms were mainly enriched in macrophage activation involved in immune response, regulation of granulocyte macrophage colony-stimulating factor production and granulacyte macrophage colony- stimulating factor production. And the KEGG terms were mainly enriched in nicotine addiction, Fc epsilon RI signaling pathway and B cell receptor signaling pathway (**Figure 6J**).

### 3.11 Identification of macrophage module genes

**Figure 7:**
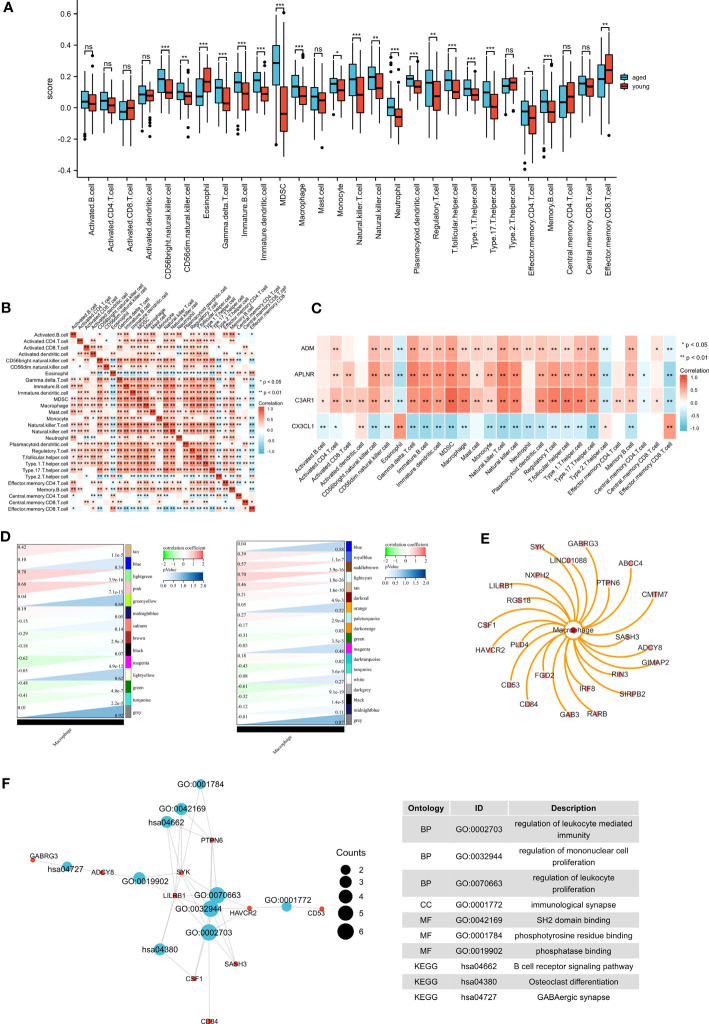
Immune infiltration analysis and identification of macrophage-related modular genes. **(A)** The ssGSEA algorithm was used to analyze the content of immune cells , *P <0.05; **P <0.01; ***P <0.001. **(B)** Correlation analysis was performed on all immune cells in the ssGSEA algorithm. **(C)** The correlations between biomarkers and immune cells. **(D)** The correlation between gene modules and macrophage. **(E)** Macrophage-related module genes. **(F)** GO and KEGG pathways enriched by the macrophage-related module genes.

The results of ssGSEA showed that macrophages were altered in the aged group (**Figure 7A**). We demonstrated the immune cell heat map (**Figure 7B**), and exhibited the correlations between biomarkers and immune cells (**Figure 7C**). We then integrated the results of WGCNA and immuno-infiltration analyses to identify the macrophage module genes. Interestingly, our findings revealed a significant association between macrophages and the lightcyan module and lightgreen module (**Figure 7D**). we then identified 21 genes that are associated with macrophages (**Figure 7E**). To further understand their function, we conducted functional enrichment analysis on these genes (**Figure 7F**). GO terms were mainly enriched in regulation of leukocyte mediated immunity, regulation of mononuclear cell proliferation and regulation of leukocyte proliferation. And the KEGG terms were mainly enriched in B cell receptor signaling pathway, Osteoclast differentiation and GABAergic synapse.

## 4 Discussion

Brain aging is a crucial aspect of organism aging that involves the gradual decline of the brain’s morphological structure and physiological function due to aging (14). This decline manifests as varying degrees of damage and impairment to brain function, and some elderly individuals may develop mild cognitive dysfunction or even dementia, creating significant pressure for families and society and becoming a severe medical and social issue (15). Therefore, exploring the mechanisms of brain aging, identifying new therapeutic targets, and slowing down the process of brain aging are of significant social importance (16). Our study conducted a bioinformatics analysis to explore the key mechanisms of brain aging across different brain regions. The results showed that the DEGs shared by four brain regions were significantly enriched in terms related to inflammatory response. During the natural aging process, an imbalance between pro- and anti-inflammatory factors leads to a state of low-grade, chronic, systemic inflammatory response, which progressively increases and is referred to as inflammaging (17). Studies have shown that inflammaging can have a significant impact on brain function, primarily through increased expression of inflammatory factors, elevated levels of oxidative stress, and disruption of the blood-brain barrier (18). These mechanisms can lead to neuronal and glial cell death or impaired function, ultimately resulting in brain atrophy and decreased function.

We performed drug prediction for inflammatory response - related DEGs and found that insulin and celecoxib can target multiple inflammaging genes simultaneously and is closely associated with inflammaging in the brain. Aging is associated with a sustained decrease in insulin release (19). In brain, insulin can promote cell survival and regulates learning and memory. But the brain can become insulin-resistant during aging due to reduced insulin expression, delivery, and binding affinity to its receptors (20). Improving insulin resistance in the brain may delay the brain aging process (21, 22). Celecoxib, a nonsteroidal drug used to treat pain and inflammation, has been found to extend life span and delay aging (23). Its mechanism of action may be related to its ability to prevent inflammaging-induced cell senescence (24).

Multiple machine learning techniques were utilized to identify four biomarkers. CX3CL1, being the exclusive member of the CX3C family of chemokines, plays a vital role in the maintenance of cognitive function (25). Exogenous administration of CX3CL1 to aged rats was found to reverse the decrease in neuroregenerative function induced by aging (26). Adrenomedullin (ADM) is involved in various physiological functions such as vasodilation, hormone regulation, and angiogenesis, and its neuroprotective properties have been demonstrated. Studies have also shown that ADM is an important biomarker of cardiovascular aging (27, 28). The C3a receptor encoded by the C3AR1 gene is a G protein-coupled receptor that is mainly distributed in neurons and glial cells of the central nervous system. The complement C3a/C3a receptor (C3a/C3aR) axis has been found to influence normal brain aging and disease progression (29). The expression of APLNR mRNA is notably high in various regions of the human brain tissue, including the cortex, caudate nucleus, corpus callosum, and hippocampus, suggesting that APLNR performs crucial functions in these brain regions (30). Mounting evidence indicates that the Apelin/APLNR system can impede cell death and facilitate neovascularization, thus enhancing brain function (31).

Single-gene GSVA analysis identified four biomarkers primarily enriched in Parkinson’s disease and the Notch signaling pathway. In the aging process, the brain is among the first organs to show signs of aging, and inflammaging of the brain can lead to various neurodegenerative diseases, including Alzheimer’s disease, Parkinson’s disease, and Huntington’s disease (32–34). Recent research suggests a close relationship between the Notch signaling pathway and cellular senescence. Inhibition of this pathway has been shown to trigger the onset of cellular senescence (35). Such as, decreased Notch signaling pathway activity is thought to be a key factor in the aging of skeletal muscle cells in older individuals (36).

Cellular senescence is the root cause of human body aging, and through analysis of single cell sequencing data, it has been found that endothelial cells are significantly reduced in older individuals compared to younger individuals. Endothelial cells are crucial for maintaining brain function by supporting neurons, preserving the blood-brain barrier, and participating in immune and inflammatory responses within the brain. Studies by Nation et al. have suggested that blood-brain barrier damage is an early indicator of cognitive decline during aging (37). Additionally, Montagne et al. have shown that blood-brain barrier damage begins to appear at the age of 55 and gradually increases with age, indicating a positive correlation between the severity of blood-brain barrier damage and age (38). Several studies have confirmed that a large portion of blood-brain barrier damage is caused by inflammation. Specifically, inflammatory factors can lead to endothelial cell damage in the brain, which increases the permeability of the blood-brain barrier. We subsequently examine the roles of biomarkers at the single-cell level. And only the levels of CX3CL1 were found to decline in inhibitory neurons, excitatory neurons, and astrocytes in older brains, while the coefficient of variation of CX3CL1 increased with advancing age. These findings suggest that CX3CL1 could serve as a critical biomarker of inflammaging in the brain.

We subsequently conducted consensus clustering analysis to divide the aged samples into two groups and performed GSEA analysis, which revealed significant enrichment of genes in immune-related pathways between the two groups. The aging of the immune system is one of the causes of inflammaging, and innate immune cells drive inflammaging by increasing pro-inflammatory subpopulations and generating pro-inflammatory responses (17). And, macrophages, which are important players in immune surveillance and clearance of aging cells during immunosenescence, are highly susceptible to aging (39).

To further investigate the mechanisms of Inflammaging in different brain regions, we identified seven inflammaging-related genes in different brain regions. These genes were significantly enriched in macrophage immunity. Subsequently, we performed immune infiltration analysis and found that the number of macrophages was significantly increased in the aged group. The analysis showed that macrophages had a significant negative correlation with CX3CL1 and a significant positive correlation with ADM, APLNR, and C3AR1. In the brain, CX3CL1 inhibits microglial polarization towards the M1 phenotype and promotes polarization towards the M2 phenotype, thereby participating in neuroprotective effects. And low expression of CX3CL1 leads to increased migration of M1-polarized macrophages, and enhanced inflammatory response (40). Recent studies have demonstrated that exposure of macrophages to aging-associated secretory phenotypes can induce polarization towards the M1 type (41). Our study observed a decrease in CX3CL1 expression and an increase in macrophage expression in the elderly group, indicating a potential shift towards M1 polarization of macrophages.

Based on the results of immuno-infiltration analysis and the results of WGCNA analysis, we identified 21 macrophage module genes. Functional enrichment analysis suggested that macrophage module genes were significantly enriched on B-cell receptor signaling pathways and GABAergic synapse. During aging, the function and number of B cells as well as humoral immunity are altered accordingly. It was found that senescent B-cell function is also significantly reduced in the elderly population and therefore fails to produce an effective humoral immune response. In addition, different types of immune cells interact with each other, such as macrophages that can influence B-cell recruitment (42). As an inhibitory neurotransmitter in the brain, GABA has physiological functions such as anti-aging, diuretic, immune enhancement and obesity prevention. Existing studies have demonstrated that neurotransmitters can influence the fate of immune cells. GABA attenuates the macrophage-mediated inflammatory response *in vivo*, and this mechanism may be related to the fact that GABA affects macrophage differentiation (43).

Our study highlights the significant role of inflammaging and immunosenescence in brain aging, which exhibit synergistic effects. Specifically, we found that inflammaging can impact macrophages in the brain, thereby aggravating the aging process. Moreover, we identified CX3CL1 as a crucial biomarker of inflammaging in the brain, which can modulate macrophage polarization. Therefore, we propose that CX3CL1-mediated macrophage polarization can exacerbate inflammaging and immunosenescence of the brain, and targeting CX3CL1 may delay brain aging by inhibiting these processes.

While our study provides valuable insights into brain aging across different regions, there are some limitations that need to be addressed. Firstly, increasing the sample size could improve the accuracy of brain aging evaluation and prediction. Secondly, the biomarkers of brain inflammaging identified in our study could be further verified *in vitro* using different cell lines, providing practical evidence for clinical targeted therapy. Thirdly, the use of animal models that knock down or overexpress CX3CL1 or *in vitro* cell molecular experiments could provide substantial evidence. Although recent progress has been made in understanding the relationship between brain inflammaging and CX3CL1 levels, further exploration is necessary. Furthermore, exploring the correlation between macrophage polarization and brain inflammaging warrants further investigation. In addition, exploring the key molecular characteristics of longevity populations is essential as it can offer valuable insights into the biological processes and mechanisms associated with longevity. Hence, more comprehensive *in vivo* and *in vitro* studies will be necessary to validate these findings in the future.

## 5 Conclusion

This study offers significant insights into the potential mechanisms involved in the onset and progression of inflammaging in the brain. Specifically, our findings highlight the potential of CX3CL1 as a target for delaying brain inflammation and immune senescence, which may be related to its ability to influence macrophage polarization. Although additional research is required to validate these findings, our study provides novel molecular perspectives that could contribute to the advancement of strategies aimed at slowing down the aging process in the brain.

## Data availability statement

The original contributions presented in the study are included in the article/**Supplementary Material**, further inquiries can be directed to the corresponding author/s.

## Author contributions

ZD and LY designed this study, collected the data and performed the bioinformatic and statistical analysis, figure visualization, and manuscript writing. TZ, YJ and YW conducted animal and RT-PCR experiments. LY and ZZ revised the manuscript. All authors listed have made a substantial, direct, and intellectual contribution to the work and approved it for publication.
