# Supplementary material for: Integrated bioinformatic analysis and experimental validation for exploring the key molecular of brain inflammaging
Source: Front Immunol. 2023 Jul 10;14:1213351. doi: 10.3389/fimmu.2023.1213351 (PMC10363601; doi:10.3389/fimmu.2023.1213351)
Supplement: Supplementary file 1 [file DataSheet_1.zip › R.package.docx]

install.packages("glmnet")

set.seed(123)

library(glmnet)

rt=read.table(inputFile, header=T, sep="\t", check.names=F, row.names=1)

rt=t(rt)

x=as.matrix(rt)

y=gsub("(.*)\\_(.*)", "\\2", row.names(rt))

fit=glmnet(x, y, family = "binomial", alpha=1)

cvfit=cv.glmnet(x, y, family="binomial", alpha=1,type.measure='deviance',nfolds = 10)

pdf(file="cvfit.pdf",width=6,height=5.5)

plot(cvfit)

dev.off()

coef=coef(fit, s = cvfit$lambda.min)

index=which(coef != 0)

lassoGene=row.names(coef)[index]

lassoGene=lassoGene[-1]

write.table(lassoGene, file="LASSO.gene.txt", sep="\t", quote=F, row.names=F, col.names=F)

install.packages("randomForest")

library(randomForest)

set.seed(123456)

data=read.table(inputFile, header=T, sep="\t", check.names=F, row.names=1)

data=t(data)

group=gsub("(.*)\\_(.*)", "\\2", row.names(data))

rf=randomForest(as.factor(group)~., data=data, ntree=500)

pdf(file="forest.pdf", width=6, height=6)

plot(rf, main="Random forest", lwd=2)

dev.off()

optionTrees=which.min(rf$err.rate[,1])

optionTrees

rf2=randomForest(as.factor(group)~., data=data, ntree=optionTrees)

importance=importance(x=rf2)

pdf(file="geneImportance.pdf", width=6.2, height=5.8)

varImpPlot(rf2, main="")

dev.off()

rfGenes=importance[order(importance[,"MeanDecreaseGini"], decreasing = TRUE),]

rfGenes=names(rfGenes[rfGenes>2])

#rfGenes=names(rfGenes[1:30])

write.table(rfGenes, file="rfGenes.txt", sep="\t", quote=F, col.names=F, row.names=F)

sigExp=t(data[,rfGenes])

sigExpOut=rbind(ID=colnames(sigExp),sigExp)

write.table(sigExpOut, file="rfGeneExp.txt", sep="\t", quote=F, col.names=F)

if (!requireNamespace("BiocManager", quietly = TRUE))

install.packages("BiocManager")

BiocManager::install("limma")

library(limma)

expFile="rfGeneExp.txt"

diffFile="diff.txt"

setwd("C:\\biowolf\\neuralDiagnostic\\14.geneScore")

rt=read.table(expFile, header=T, sep="\t", check.names=F)

rt=as.matrix(rt)

rownames(rt)=rt[,1]

exp=rt[,2:ncol(rt)]

dimnames=list(rownames(exp),colnames(exp))

data=matrix(as.numeric(as.matrix(exp)),nrow=nrow(exp),dimnames=dimnames)

data=avereps(data)

diffRT=read.table(diffFile, header=T, sep="\t", check.names=F, row.names=1)

diffRT=diffRT[row.names(data),]

dataUp=data[diffRT[,"logFC"]>0,]

dataDown=data[diffRT[,"logFC"]<0,]

dataUp2=t(apply(dataUp,1,function(x)ifelse(x>median(x),1,0)))

dataDown2=t(apply(dataDown,1,function(x)ifelse(x>median(x),0,1)))

outTab=rbind(dataUp2, dataDown2)

outTab=rbind(id=colnames(outTab), outTab)

write.table(outTab, file="geneScore.txt", sep="\t", quote=F, col.names=F)

install.packages("neuralnet")

install.packages("NeuralNetTools")

library(neuralnet)

library(NeuralNetTools)

set.seed(12345678)

inputFile="geneScore.txt"

data=read.table(inputFile, header=T, sep="\t", check.names=F, row.names=1)

data=as.data.frame(t(data))

group=gsub("(.*)\\_(.*)", "\\2", row.names(data))

data$con=ifelse(group=="con", 1, 0)

data$treat=ifelse(group=="treat", 1, 0)

fit=neuralnet(con+treat~., data, hidden=5)

fit$result.matrix

fit$weight

pdf(file="neuralnet.pdf", width=9, height=7)

plotnet(fit)

dev.off()

net.predict=compute(fit, data)$net.result

net.prediction=c("con", "treat")[apply(net.predict, 1, which.max)]

predict.table=table(group, net.prediction)

predict.table

conAccuracy=predict.table[1,1]/(predict.table[1,1]+predict.table[1,2])

treatAccuracy=predict.table[2,2]/(predict.table[2,1]+predict.table[2,2])

paste0("Con accuracy: ", sprintf("%.3f", conAccuracy))

paste0("Treat accuracy: ", sprintf("%.3f", treatAccuracy))

colnames(net.predict)=c("con", "treat")

outTab=rbind(id=colnames(net.predict), net.predict)

write.table(outTab, file="neural.predict.txt", sep="\t", quote=F, col.names=F)

install.packages("pROC")

library(pROC)

inputFile="neural.predict.txt"

rt=read.table(inputFile, header=T, sep="\t", check.names=F, row.names=1)

y=gsub("(.*)\\_(.*)", "\\2", row.names(rt))

y=ifelse(y=="con", 0, 1)

roc1=roc(y, as.numeric(rt[,2]))

ci1=ci.auc(roc1, method="bootstrap")

ciVec=as.numeric(ci1)

pdf(file="ROC.pdf", width=5, height=5)

plot(roc1, print.auc=TRUE, col="red", legacy.axes=T, main="Train group")

text(0.39, 0.43, paste0("95% CI: ",sprintf("%.03f",ciVec[1]),"-",sprintf("%.03f",ciVec[3])), col="red")

dev.off()

if (!requireNamespace("BiocManager", quietly = TRUE))

install.packages("BiocManager")

BiocManager::install("limma")

library(limma)

expFile="GSE130928.txt"

conFile="GSE130928_s1.txt"

treatFile="GSE130928_s2.txt"

diffFile="diff.txt"

rt=read.table(expFile, header=T, sep="\t", check.names=F)

rt=as.matrix(rt)

rownames(rt)=rt[,1]

exp=rt[,2:ncol(rt)]

dimnames=list(rownames(exp), colnames(exp))

data=matrix(as.numeric(as.matrix(exp)), nrow=nrow(exp), dimnames=dimnames)

rt=avereps(data)

qx=as.numeric(quantile(rt, c(0, 0.25, 0.5, 0.75, 0.99, 1.0), na.rm=T))

LogC=( (qx[5]>100) || ( (qx[6]-qx[1])>50 && qx[2]>0) )

if(LogC){

rt[rt<0]=0

rt=log2(rt+1)}

data=normalizeBetweenArrays(rt)

con=read.table(conFile, header=F, sep="\t", check.names=F)

treat=read.table(treatFile, header=F, sep="\t", check.names=F)

conData=data[,as.vector(con[,1])]

treatData=data[,as.vector(treat[,1])]

data=cbind(conData, treatData)

conNum=ncol(conData)

treatNum=ncol(treatData)

Type=c(rep("con",conNum), rep("treat",treatNum))

colnames(data)=paste0(colnames(data),"_",Type)

diffRT=read.table(diffFile, header=T, sep="\t", check.names=F, row.names=1)

sameGene=intersect(row.names(data), row.names(diffRT))

diffRT=diffRT[sameGene,]

data=data[sameGene,]

dataUp=data[diffRT[,"logFC"]>0,]

dataDown=data[diffRT[,"logFC"]<0,]

dataUp2=t(apply(dataUp,1,function(x)ifelse(x>median(x),1,0)))

dataDown2=t(apply(dataDown,1,function(x)ifelse(x>median(x),0,1)))

outTab=rbind(dataUp2, dataDown2)

outTab=rbind(id=colnames(outTab), outTab)

write.table(outTab, file="testGeneScore.txt", sep="\t", quote=F, col.names=F)

set.seed(12345678)

trainFile="geneScore.txt"

testFile="testGeneScore.txt"

data=read.table(trainFile, header=T, sep="\t", check.names=F, row.names=1)

data=as.data.frame(t(data))

group=gsub("(.*)\\_(.*)", "\\2", row.names(data))

data$con=ifelse(group=="con", 1, 0)

data$treat=ifelse(group=="treat", 1, 0)

fit=neuralnet(con+treat~., data, hidden = 5)

data2=read.table(testFile, header=T, sep="\t", check.names=F, row.names=1)

data2=t(data2)

group2=gsub("(.*)\\_(.*)", "\\2", row.names(data2))

sameGene=intersect(colnames(data), colnames(data2))

data2=data2[,sameGene]

net.predict=compute(fit, data2)$net.result

net.prediction = c("con", "treat")[apply(net.predict, 1, which.max)]

predict.table = table(group2, net.prediction)

predict.table

conAccuracy=predict.table[1,1]/(predict.table[1,1]+predict.table[1,2])

treatAccuracy=predict.table[2,2]/(predict.table[2,1]+predict.table[2,2])

paste0("Con accuracy: ", sprintf("%.3f", conAccuracy))

paste0("Treat accuracy: ", sprintf("%.3f", treatAccuracy))

colnames(net.predict)=c("con", "treat")

outTab=rbind(id=colnames(net.predict), net.predict)

write.table(outTab, file="test.neuralPredict.txt", sep="\t", quote=F, col.names=F)

install.packages("pROC")

library(pROC)

inputFile="test.neuralPredict.txt"

rt=read.table(inputFile, header=T, sep="\t", check.names=F, row.names=1)

y=gsub("(.*)\\_(.*)", "\\2", row.names(rt))

y=ifelse(y=="con", 0, 1)

roc1=roc(y, as.numeric(rt[,2]))

ci1=ci.auc(roc1, method="bootstrap")

ciVec=as.numeric(ci1)

pdf(file="ROC.pdf", width=5, height=5)

plot(roc1, print.auc=TRUE, col="red", legacy.axes=T, main="Test group")

text(0.39, 0.43, paste0("95% CI: ",sprintf("%.03f",ciVec[1]),"-",sprintf("%.03f",ciVec[3])), col="red")

dev.off()
