# Supplementary material for: Integrated bioinformatic analysis and experimental validation for exploring the key molecular of brain inflammaging
Source: Front Immunol. 2023 Jul 10;14:1213351. doi: 10.3389/fimmu.2023.1213351 (PMC10363601; doi:10.3389/fimmu.2023.1213351)
Supplement: Supplementary file 3 [file DataSheet_3.zip › R.package.docx]

if (!requireNamespace("BiocManager", quietly = TRUE))

install.packages("BiocManager")

BiocManager::install("limma")

BiocManager::install("GSEABase")

BiocManager::install("GSVA")

install.packages("ggpubr")

library(reshape2)

library(ggpubr)

library(limma)

library(GSEABase)

library(GSVA)

gene="SOCS1"

expFile="normalize.txt"

gmtFile="c2.cp.kegg.symbols.gmt"

rt=read.table(expFile, header=T, sep="\t", check.names=F)

rt=as.matrix(rt)

rownames(rt)=rt[,1]

exp=rt[,2:ncol(rt)]

dimnames=list(rownames(exp),colnames(exp))

data=matrix(as.numeric(as.matrix(exp)),nrow=nrow(exp),dimnames=dimnames)

data=avereps(data)

group=gsub("(.*)\\_(.*)", "\\2", colnames(data))

data=data[,group=="Treat",drop=F]

geneSets=getGmt(gmtFile, geneIdType=SymbolIdentifier())

ssgseaScore=gsva(data, geneSets, method='ssgsea', kcdf='Gaussian', abs.ranking=TRUE)

normalize=function(x){

return((x-min(x))/(max(x)-min(x)))}

ssgseaScore=normalize(ssgseaScore)

ssgseaScore=ssgseaScore[order(apply(ssgseaScore,1,sd),decreasing=T),]

ssgseaScore=ssgseaScore[1:50,]

lowName=colnames(data)[data[gene,]<median(data[gene,])]

highName=colnames(data)[data[gene,]>=median(data[gene,])]

lowScore=ssgseaScore[,lowName]

highScore=ssgseaScore[,highName]

data=cbind(lowScore, highScore)

conNum=ncol(lowScore)

treatNum=ncol(highScore)

Type=c(rep("Control",conNum), rep("Treat",treatNum))

outTab=data.frame()

for(i in row.names(data)){

test=t.test(data[i,] ~ Type)

pvalue=test$p.value

t=test$statistic

Sig=ifelse(pvalue>0.05, "Not", ifelse(t>0,"Up","Down"))

outTab=rbind(outTab, cbind(Pathway=i, t, pvalue, Sig))

}

pdf(file="barplot.pdf", width=12, height=9)

outTab$t=as.numeric(outTab$t)

outTab$Sig=factor(outTab$Sig, levels=c("Down", "Not", "Up"))

gg1=ggbarplot(outTab, x="Pathway", y="t", fill = "Sig", color = "white",

palette=c("green3","grey","red3"), sort.val = "asc", sort.by.groups = T,

rotate=TRUE, legend="right", title=gene,

xlab="Term", ylab="t value of GSVA score", legend.title="Group", x.text.angle=60)

print(gg1)

dev.off()
