# Supplementary figures and images for: Integrated bioinformatic analysis and experimental validation for exploring the key molecular of brain inflammaging
Source: Front Immunol. 2023 Jul 10;14:1213351. doi: 10.3389/fimmu.2023.1213351 (PMC10363601; doi:10.3389/fimmu.2023.1213351)

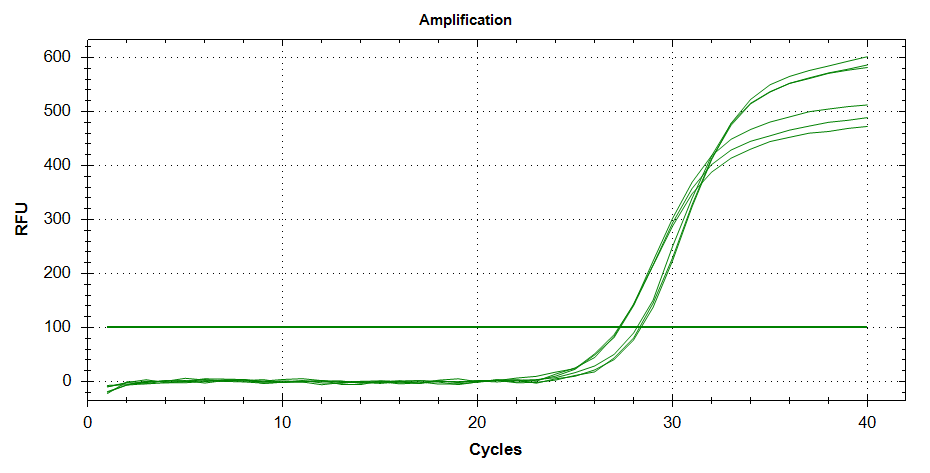

Supplement: Supplementary file 4 [file DataSheet_4.zip › Amplification curve/ADM.png]

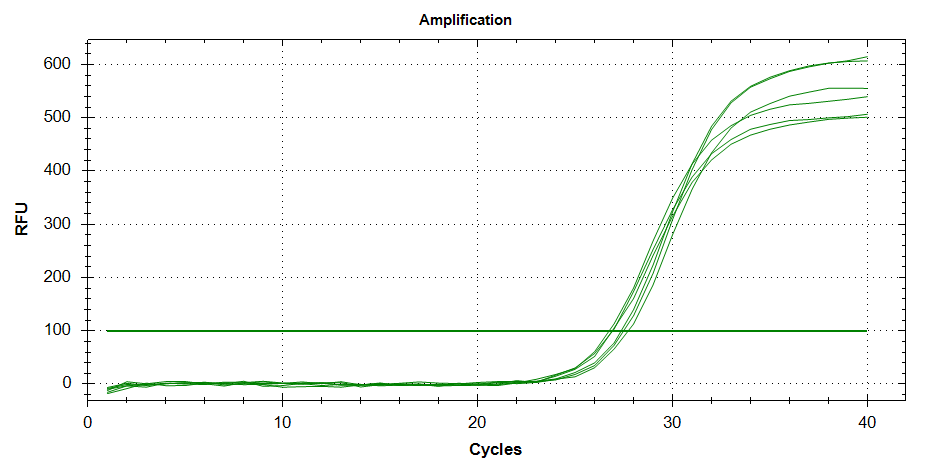

Supplement: Supplementary file 4 [file DataSheet_4.zip › Amplification curve/APLNR.png]

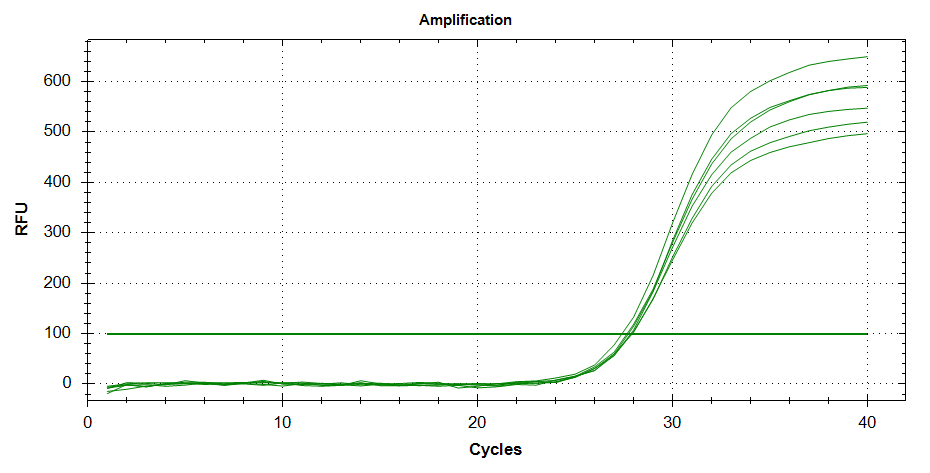

Supplement: Supplementary file 4 [file DataSheet_4.zip › Amplification curve/C3AR1.png]

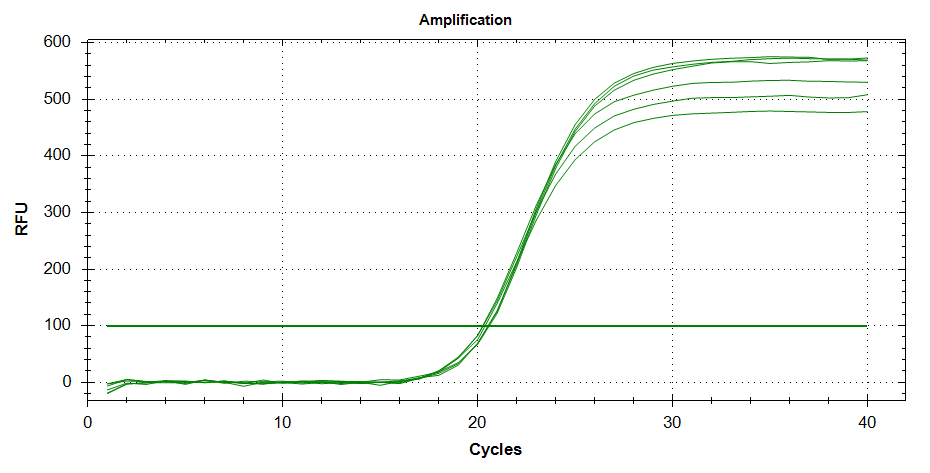

Supplement: Supplementary file 4 [file DataSheet_4.zip › Amplification curve/CX3CL1.png]

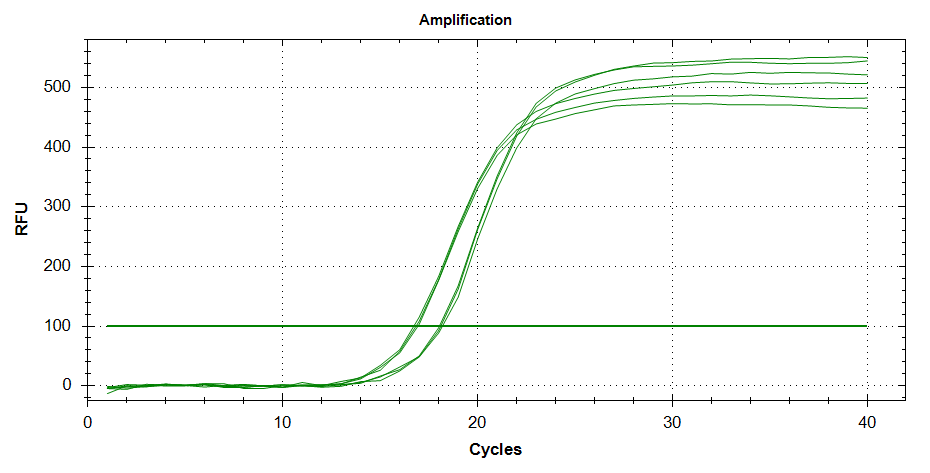

Supplement: Supplementary file 4 [file DataSheet_4.zip › Amplification curve/GAPDH.png]

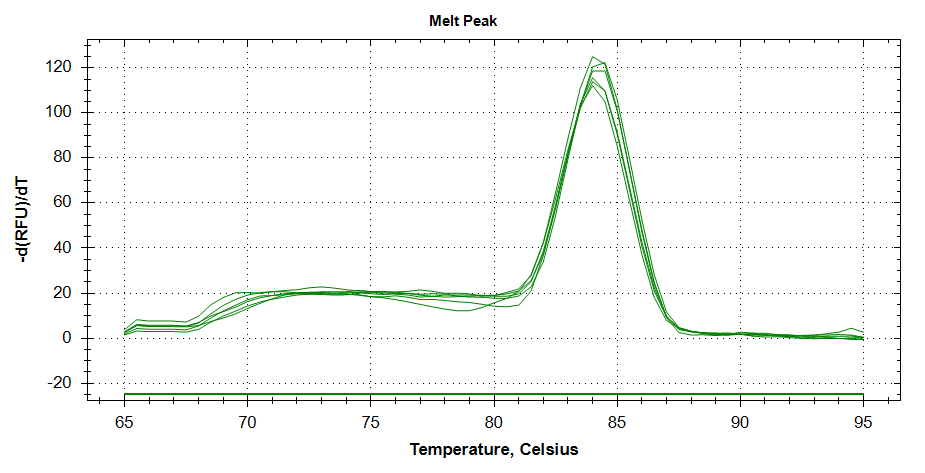

Supplement: Supplementary file 4 [file DataSheet_4.zip › Melting curve/ADM.png]

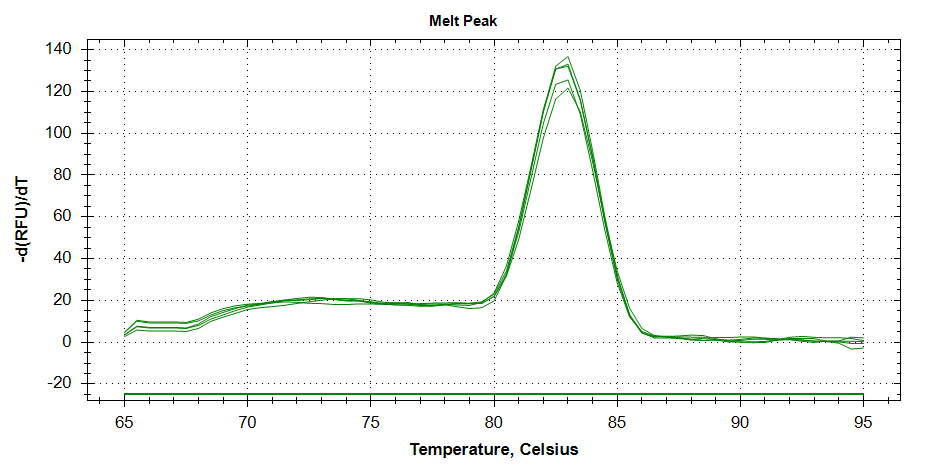

Supplement: Supplementary file 4 [file DataSheet_4.zip › Melting curve/APLNR.png]

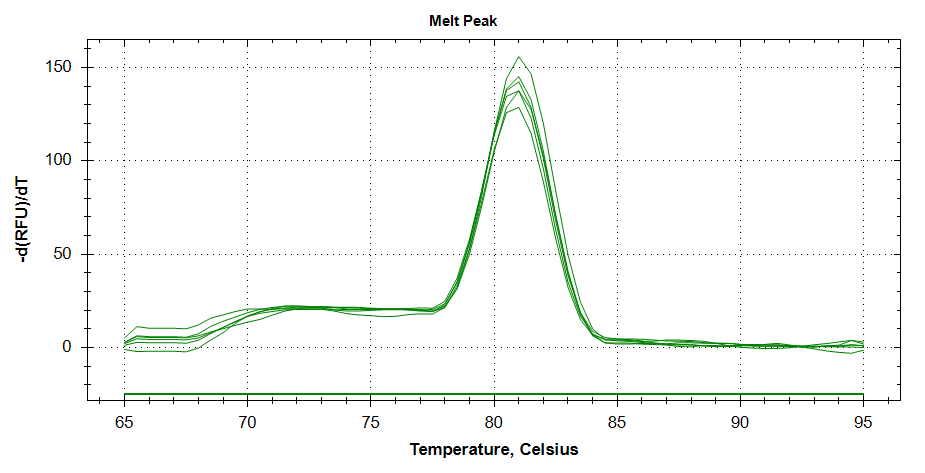

Supplement: Supplementary file 4 [file DataSheet_4.zip › Melting curve/C3AR1.png]

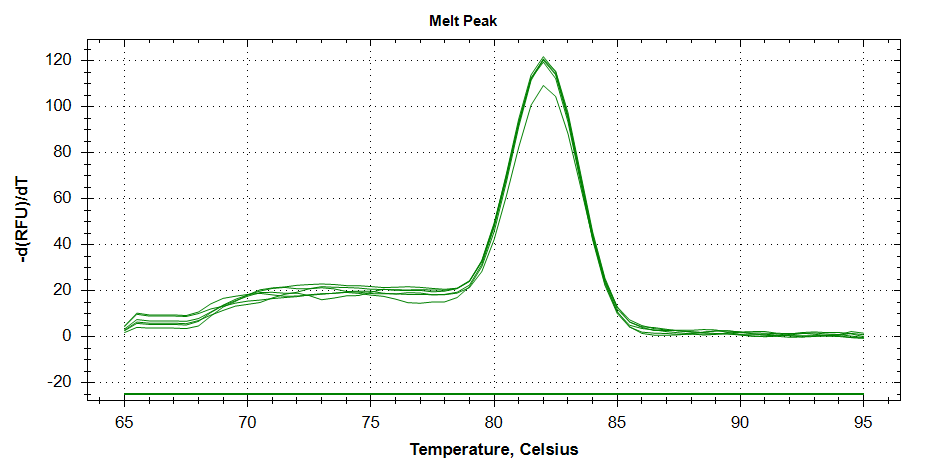

Supplement: Supplementary file 4 [file DataSheet_4.zip › Melting curve/CX3CL1.png]

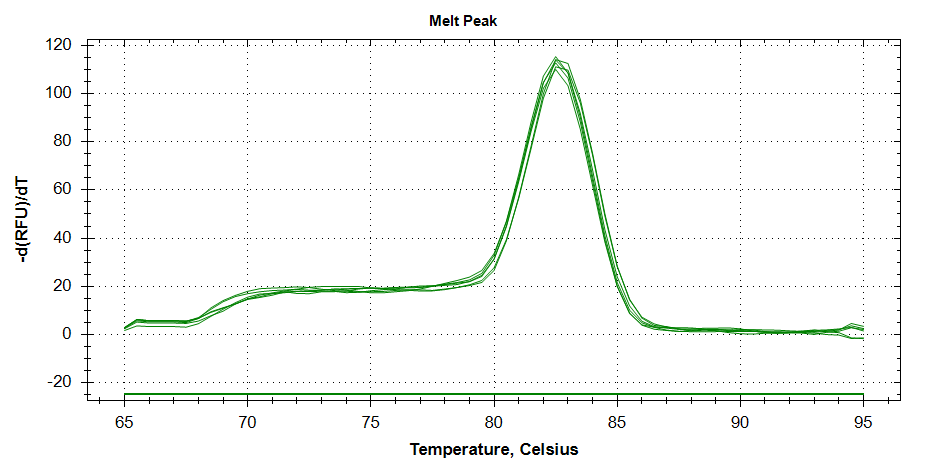

Supplement: Supplementary file 4 [file DataSheet_4.zip › Melting curve/GAPDH.png]
