# Supplementary material for: Integrated bioinformatic analysis and experimental validation for exploring the key molecular of brain inflammaging
Source: Front Immunol. 2023 Jul 10;14:1213351. doi: 10.3389/fimmu.2023.1213351 (PMC10363601; doi:10.3389/fimmu.2023.1213351)
Supplement: Supplementary file 5 [file DataSheet_5.zip › R.package.docx]

if (!requireNamespace("BiocManager", quietly = TRUE))

install.packages("BiocManager")

BiocManager::install("ConsensusClusterPlus")

library(ConsensusClusterPlus)

expFile="diffGeneExp.txt"

workDir="C:\\biowolf\\geoCRG\\14.cluster"

setwd(workDir)

data=read.table(expFile, header=T, sep="\t", check.names=F, row.names=1)

data=as.matrix(data)

group=sapply(strsplit(colnames(data),"\\_"), "[", 2)

data=data[,group=="Treat"]

maxK=9

results=ConsensusClusterPlus(data,

maxK=maxK,

reps=50,

pItem=0.8,

pFeature=1,

title=workDir,

clusterAlg="km",

distance="euclidean",

seed=123456,

plot="png")

calcICL(results, title="consensusScore", plot="png")

clusterNum=2

cluster=results[[clusterNum]][["consensusClass"]]

cluster=as.data.frame(cluster)

colnames(cluster)=c("Cluster")

cluster$Cluster=paste0("C", cluster$Cluster)

outTab=cbind(t(data), cluster)

outTab=rbind(ID=colnames(outTab), outTab)

write.table(outTab, file="cluster.txt", sep="\t", quote=F, col.names=F)

library(limma)

library(ggplot2)

clusterFile="cluster.txt"

setwd("C:\\biowolf\\geoCRG\\16.PCA")

rt=read.table(clusterFile, header=T, sep="\t", check.names=F, row.names=1)

data=rt[,1:(ncol(rt)-1),drop=F]

Cluster=as.vector(rt[,ncol(rt)])

data.pca=prcomp(data)

pcaPredict=predict(data.pca)

PCA=data.frame(PC1=pcaPredict[,1], PC2=pcaPredict[,2], Cluster=Cluster)

PCA.mean=aggregate(PCA[,1:2], list(Cluster=PCA$Cluster), mean)

bioCol=c("#0066FF","#FF0000","#FF9900","#6E568C","#7CC767","#223D6C","#D20A13","#FFD121","#088247","#11AA4D")

crgCluCol=bioCol[1:length(levels(factor(Cluster)))]

veganCovEllipse<-function (cov, center = c(0, 0), scale = 1, npoints = 100) {

theta <- (0:npoints) * 2 * pi/npoints

Circle <- cbind(cos(theta), sin(theta))

t(center + scale * t(Circle %*% chol(cov)))

}

df_ell <- data.frame()

for(g in levels(factor(PCA$Cluster))){

df_ell <- rbind(df_ell, cbind(as.data.frame(with(PCA[PCA$Cluster==g,],

veganCovEllipse(cov.wt(cbind(PC1,PC2),

wt=rep(1/length(PC1),length(PC1)))$cov,

center=c(mean(PC1),mean(PC2))))), Cluster=g))

}

pdf(file="PCA.pdf", width=6.5, height=5)

ggplot(data = PCA, aes(PC1, PC2)) + geom_point(aes(color = Cluster)) +

scale_colour_manual(name="Cluster", values =crgCluCol)+

theme_bw()+

theme(plot.margin=unit(rep(1.5,4),'lines'))+

geom_path(data=df_ell, aes(x=PC1, y=PC2, colour=Cluster), size=1, linetype=2)+

annotate("text",x=PCA.mean$PC1, y=PCA.mean$PC2, label=PCA.mean$Cluster, cex=7)+

theme(panel.grid.major = element_blank(), panel.grid.minor = element_blank())

dev.off()

#if (!requireNamespace("BiocManager", quietly = TRUE))

# install.packages("BiocManager")

#BiocManager::install("limma")

#install.packages("pheatmap")

#install.packages("reshape2")

#install.packages("ggpubr")

library(limma)

library(pheatmap)

library(reshape2)

library(ggpubr)

clusterFile="cluster.txt"

setwd("C:\\biowolf\\geoCRG\\15.clusterHeatmap")

rt=read.table(clusterFile, header=T, sep="\t", check.names=F, row.names=1)

rt=rt[order(rt$Cluster),]

data=t(rt[,1:(ncol(rt)-1),drop=F])

Type=rt[,ncol(rt),drop=F]

bioCol=c("#0066FF","#FF0000","#FF9900","#6E568C","#7CC767","#223D6C","#D20A13","#FFD121","#088247","#11AA4D")

ann_colors=list()

crgCluCol=bioCol[1:length(levels(factor(Type$Cluster)))]

names(crgCluCol)=levels(factor(Type$Cluster))

ann_colors[["Cluster"]]=crgCluCol

pdf("heatmap.pdf", width=7, height=4.5)

pheatmap(data,

annotation=Type,

annotation_colors = ann_colors,

color = colorRampPalette(c(rep("blue",2), "white", rep("red",2)))(100),

cluster_cols =F,

cluster_rows =T,

scale="row",

show_colnames=F,

show_rownames=T,

fontsize=7,

fontsize_row=7,

fontsize_col=7)

dev.off()

data=melt(rt, id.vars=c("Cluster"))

colnames(data)=c("Cluster", "Gene", "Expression")

p=ggboxplot(data, x="Gene", y="Expression", color = "Cluster",

xlab="",

ylab="Gene expression",

legend.title="Cluster",

palette = crgCluCol,

width=0.8,

add="point")

p=p+rotate_x_text(60)

p1=p+stat_compare_means(aes(group=Cluster),

symnum.args=list(cutpoints = c(0, 0.001, 0.01, 0.05, 1), symbols = c("***", "**", "*", " ")),

label = "p.signif")

pdf(file="boxplot.pdf", width=7, height=5)

print(p1)

dev.off()

if (!requireNamespace("BiocManager", quietly = TRUE))

install.packages("BiocManager")

BiocManager::install("limma")

BiocManager::install("org.Hs.eg.db")

BiocManager::install("DOSE")

BiocManager::install("clusterProfiler")

BiocManager::install("enrichplot")

library(limma)

library(org.Hs.eg.db)

library(clusterProfiler)

library(enrichplot)

expFile="normalize.txt"

gmtFile="c2.cp.kegg.symbols.gmt"

setwd("C:\\biowolf\\geoFRG\\15.GSEA")

rt=read.table(expFile, header=T, sep="\t", check.names=F)

rt=as.matrix(rt)

rownames(rt)=rt[,1]

exp=rt[,2:ncol(rt)]

dimnames=list(rownames(exp),colnames(exp))

data=matrix(as.numeric(as.matrix(exp)),nrow=nrow(exp),dimnames=dimnames)

data=avereps(data)

data=data[rowMeans(data)>0,]

group=gsub("(.*)\\_(.*)", "\\2", colnames(data))

data=data[,group=="Treat",drop=F]

dataL=data[,data[gene,]<median(data[gene,]),drop=F]

dataH=data[,data[gene,]>=median(data[gene,]),drop=F]

meanL=rowMeans(dataL)

meanH=rowMeans(dataH)

meanL[meanL<0.00001]=0.00001

meanH[meanH<0.00001]=0.00001

logFC=log2(meanH)-log2(meanL)

logFC=sort(logFC, decreasing=T)

genes=names(logFC)

gmt=read.gmt(gmtFile)

kk=GSEA(logFC, TERM2GENE=gmt, pvalueCutoff = 1)

kkTab=as.data.frame(kk)

kkTab=kkTab[kkTab$pvalue<0.05,]

kkTab=kkTab[kkTab$p.adjust<0.05,]

write.table(kkTab,file="GSEA.result.txt",sep="\t",quote=F,row.names = F)

termNum=6

if(nrow(kkTab)>=termNum){

showTerm=row.names(kkTab)[1:termNum]

gseaplot=gseaplot2(kk, showTerm, base_size=8, title=gene)

pdf(file="GSEA.pdf", width=7.5, height=5.5)

print(gseaplot)

dev.off()
