# Supplementary material for: Integrated bioinformatic analysis and experimental validation for exploring the key molecular of brain inflammaging
Source: Front Immunol. 2023 Jul 10;14:1213351. doi: 10.3389/fimmu.2023.1213351 (PMC10363601; doi:10.3389/fimmu.2023.1213351)
Supplement: Supplementary file 7 [file DataSheet_7.zip › R.package.docx]

#if (!requireNamespace("BiocManager", quietly = TRUE))

# install.packages("BiocManager")

#BiocManager::install("limma")

#BiocManager::install("GSEABase")

#BiocManager::install("GSVA")

#install.packages("ggpubr")

library(reshape2)

library(ggpubr)

library(limma)

library(GSEABase)

library(GSVA)

expFile="normalize.txt"

gmtFile="immune.gmt"

setwd("C:\\biowolf\\m6A\\18.ssGSEA")

rt=read.table(expFile, header=T, sep="\t", check.names=F)

rt=as.matrix(rt)

rownames(rt)=rt[,1]

exp=rt[,2:ncol(rt)]

dimnames=list(rownames(exp),colnames(exp))

data=matrix(as.numeric(as.matrix(exp)),nrow=nrow(exp),dimnames=dimnames)

data=avereps(data)

geneSets=getGmt(gmtFile, geneIdType=SymbolIdentifier())

ssgseaScore=gsva(data, geneSets, method='ssgsea', kcdf='Gaussian', abs.ranking=TRUE)

normalize=function(x){

return((x-min(x))/(max(x)-min(x)))}

ssgseaScore=normalize(ssgseaScore)

ssgseaOut=rbind(id=colnames(ssgseaScore), ssgseaScore)

write.table(ssgseaOut,file="ssGSEA.result.txt",sep="\t",quote=F,col.names=F)

cluster=read.table(clusterFile, header=T, sep="\t", check.names=F, row.names=1)

ssgseaScore=t(ssgseaScore)

sameSample=intersect(row.names(ssgseaScore), row.names(cluster))

ssgseaScore=ssgseaScore[sameSample,,drop=F]

cluster=cluster[sameSample,"m6Acluster",drop=F]

scoreCluster=cbind(ssgseaScore, cluster)

data=melt(scoreCluster, id.vars=c("m6Acluster"))

colnames(data)=c("m6Acluster", "Immune", "Fraction")

bioCol=c("#0066FF","#FF0000","#FF9900","#6E568C","#7CC767","#223D6C","#D20A13","#FFD121","#088247","#11AA4D")

bioCol=bioCol[1:length(levels(factor(data[,"m6Acluster"])))]

p=ggboxplot(data, x="Immune", y="Fraction", color="m6Acluster",

xlab="",

ylab="Immune infiltration",

legend.title="m6Acluster",

palette=bioCol)

p=p+rotate_x_text(50)

pdf(file="boxplot.pdf", width=8, height=6)

p+stat_compare_means(aes(group=m6Acluster),symnum.args=list(cutpoints = c(0, 0.001, 0.01, 0.05, 1), symbols = c("***", "**", "*", "")),label = "p.signif")

dev.off()

if (!requireNamespace("BiocManager", quietly = TRUE))

install.packages("BiocManager")

BiocManager::install(c("GO.db", "preprocessCore", "impute","limma"))

install.packages(c("matrixStats", "Hmisc", "foreach", "doParallel", "fastcluster", "dynamicTreeCut", "survival"))

install.packages("WGCNA")

library(limma)

library(WGCNA)

expFile=" CIBERSORT.txt"

immFile=" Gene expression.txt"

setwd("C:\\biowolf\\immWGCNA\\14.immuneWGCNA")

rt=read.table(expFile, header=T, sep="\t", check.names=F)

rt=as.matrix(rt)

rownames(rt)=rt[,1]

exp=rt[,2:ncol(rt)]

dimnames=list(rownames(exp),colnames(exp))

data=matrix(as.numeric(as.matrix(exp)), nrow=nrow(exp), dimnames=dimnames)

data=avereps(data)

group=sapply(strsplit(colnames(data),"\\-"), "[", 4)

group=sapply(strsplit(group,""), "[", 1)

group=gsub("2", "1", group)

data=data[,group==0,drop=F]

data=log2(data+1)

data=data[rowMeans(data)>0.2,]

data=data[apply(data,1,sd)>0.2,]

datExpr0=t(data)

gsg = goodSamplesGenes(datExpr0, verbose = 3)

if (!gsg$allOK){

# Optionally, print the gene and sample names that were removed:

if (sum(!gsg$goodGenes)>0)

printFlush(paste("Removing genes:", paste(names(datExpr0)[!gsg$goodGenes], collapse = ", ")))

if (sum(!gsg$goodSamples)>0)

printFlush(paste("Removing samples:", paste(rownames(datExpr0)[!gsg$goodSamples], collapse = ", ")))

# Remove the offending genes and samples from the data:

datExpr0 = datExpr0[gsg$goodSamples, gsg$goodGenes]

}

sampleTree = hclust(dist(datExpr0), method = "average")

pdf(file = "1_sample_cluster.pdf", width = 12, height = 9)

par(cex = 0.6)

par(mar = c(0,4,2,0))

plot(sampleTree, main = "Sample clustering to detect outliers", sub="", xlab="", cex.lab = 1.5, cex.axis = 1.5, cex.main = 2)

abline(h = 10000, col = "red")

dev.off()

clust = cutreeStatic(sampleTree, cutHeight = 10000, minSize = 10)

table(clust)

keepSamples = (clust==1)

datExpr0 = datExpr0[keepSamples, ]

enableWGCNAThreads()

powers = c(1:20)

sft = pickSoftThreshold(datExpr0, powerVector = powers, verbose = 5)

pdf(file="2_scale_independence.pdf",width=9,height=5)

par(mfrow = c(1,2))

cex1 = 0.9

plot(sft$fitIndices[,1], -sign(sft$fitIndices[,3])*sft$fitIndices[,2],

xlab="Soft Threshold (power)",ylab="Scale Free Topology Model Fit,signed R^2",type="n",

main = paste("Scale independence"));

text(sft$fitIndices[,1], -sign(sft$fitIndices[,3])*sft$fitIndices[,2],

labels=powers,cex=cex1,col="red");

abline(h=0.90,col="red")

plot(sft$fitIndices[,1], sft$fitIndices[,5],

xlab="Soft Threshold (power)",ylab="Mean Connectivity", type="n",

main = paste("Mean connectivity"))

text(sft$fitIndices[,1], sft$fitIndices[,5], labels=powers, cex=cex1,col="red")

dev.off()

softPower =sft$powerEstimate

adjacency = adjacency(datExpr0, power = softPower)

softPower

TOM = TOMsimilarity(adjacency)

dissTOM = 1-TOM

geneTree = hclust(as.dist(dissTOM), method = "average");

pdf(file="3_gene_clustering.pdf",width=12,height=9)

plot(geneTree, xlab="", sub="", main = "Gene clustering on TOM-based dissimilarity",

labels = FALSE, hang = 0.04)

dev.off()

minModuleSize=60

dynamicMods = cutreeDynamic(dendro = geneTree, distM = dissTOM,

deepSplit = 2, pamRespectsDendro = FALSE,

minClusterSize = minModuleSize);

table(dynamicMods)

dynamicColors = labels2colors(dynamicMods)

table(dynamicColors)

pdf(file="4_Dynamic_Tree.pdf",width=8,height=6)

plotDendroAndColors(geneTree, dynamicColors, "Dynamic Tree Cut",

dendroLabels = FALSE, hang = 0.03,

addGuide = TRUE, guideHang = 0.05,

main = "Gene dendrogram and module colors")

dev.off()

MEList = moduleEigengenes(datExpr0, colors = dynamicColors)

MEs = MEList$eigengenes

MEDiss = 1-cor(MEs);

METree = hclust(as.dist(MEDiss), method = "average")

pdf(file="5_Clustering_module.pdf",width=7,height=7)

plot(METree, main = "Clustering of module eigengenes",

xlab = "", sub = "")

MEDissThres = 0.25

abline(h=MEDissThres, col = "red")

dev.off()

merge = mergeCloseModules(datExpr0, dynamicColors, cutHeight = MEDissThres, verbose = 3)

mergedColors = merge$colors

mergedMEs = merge$newMEs

pdf(file="6_merged_dynamic.pdf", width = 9, height = 6)

plotDendroAndColors(geneTree, mergedColors,"Dynamic Tree Cut",

dendroLabels = FALSE, hang = 0.03,

addGuide = TRUE, guideHang = 0.05,

main = "Gene dendrogram and module colors")

dev.off()

moduleColors = mergedColors

table(moduleColors)

colorOrder = c("grey", standardColors(50))

moduleLabels = match(moduleColors, colorOrder)-1

MEs = mergedMEs

immune=read.table(immFile, header=T, sep="\t", check.names=F, row.names=1)

immune=immune[immune[,"P-value"]<0.05,]

cli=as.matrix(immune[,1:(ncol(immune)-3)])

sameSample=intersect(row.names(cli), row.names(MEs))

MEs=MEs[sameSample,,drop=F]

datTraits=cli[sameSample,,drop=F]

nGenes = ncol(datExpr0)

nSamples = nrow(datExpr0)

moduleTraitCor = cor(MEs, datTraits, use = "p")

moduleTraitPvalue = corPvalueStudent(moduleTraitCor, nSamples)

pdf(file="7_Module_trait.pdf", width=10, height=8)

textMatrix = paste(signif(moduleTraitCor, 2), "\n(",

signif(moduleTraitPvalue, 1), ")", sep = "")

dim(textMatrix) = dim(moduleTraitCor)

par(mar = c(9, 9, 3, 3))

labeledHeatmap(Matrix = moduleTraitCor,

xLabels = colnames(datTraits),

yLabels = names(MEs),

ySymbols = names(MEs),

colorLabels = FALSE,

colors = blueWhiteRed(50),

textMatrix = textMatrix,

setStdMargins = FALSE,

cex.text = 0.5,

zlim = c(-1,1),

main = paste("Module-trait relationships"))

dev.off()

probes = colnames(datExpr0)

geneInfo0 = data.frame(probes= probes,

moduleColor = moduleColors)

geneOrder =order(geneInfo0$moduleColor)

geneInfo = geneInfo0[geneOrder, ]

write.table(geneInfo, file = "module_all.txt",sep="\t",row.names=F,quote=F)

for (mod in 1:nrow(table(moduleColors))){

modules = names(table(moduleColors))[mod]

probes = colnames(datExpr0)

inModule = (moduleColors == modules)

modGenes = probes[inModule]

write.table(modGenes, file =paste0("module_",modules,".txt"),sep="\t",row.names=F,col.names=F,quote=F)

}
