# Supplementary material for: Integrated bioinformatic analysis and experimental validation for exploring the key molecular of brain inflammaging
Source: Front Immunol. 2023 Jul 10;14:1213351. doi: 10.3389/fimmu.2023.1213351 (PMC10363601; doi:10.3389/fimmu.2023.1213351)
Supplement: Supplementary file 8 [file DataSheet_8.zip › R.package.docx]

install.packages('e1071')

if (!requireNamespace("BiocManager", quietly = TRUE))

install.packages("BiocManager")

BiocManager::install("preprocessCore")

if (!requireNamespace("BiocManager", quietly = TRUE))

install.packages("BiocManager")

BiocManager::install("limma")

library("limma")

inputFile=" Gene expression.txt"

setwd("C:\\biowolf\\immWGCNA\\12.CIBERSORT")

rt=read.table(inputFile, header=T, sep="\t", check.names=F)

rt=as.matrix(rt)

rownames(rt)=rt[,1]

exp=rt[,2:ncol(rt)]

dimnames=list(rownames(exp),colnames(exp))

data=matrix(as.numeric(as.matrix(exp)),nrow=nrow(exp),dimnames=dimnames)

data=avereps(data)

data=data[rowMeans(data)>0,]

out=rbind(ID=colnames(data),data)

write.table(out,file="uniq.symbol.txt",sep="\t",quote=F,col.names=F)

source("immWGCNA12.CIBERSORT.R")

results=CIBERSORT("ref.txt", "uniq.symbol.txt", perm=1000, QN=TRUE)

install.packages("pheatmap")

install.packages("corrplot")

library(pheatmap)

library(corrplot)

pFilter=0.05

immFile="CIBERSORT.txt"

setwd("C:\\biowolf\\immWGCNA\\13.immunePlot")

immune=read.table(immFile, header=T, sep="\t", check.names=F, row.names=1)

immune=immune[immune[,"P-value"]<pFilter,]

data=as.matrix(immune[,1:(ncol(immune)-3)])

group=sapply(strsplit(row.names(data),"\\-"), "[", 4)

group=sapply(strsplit(group,""), "[", 1)

group=gsub("2", "1", group)

normalData=data[group==1,]

tumorData=data[group==0,]

normalNum=nrow(normalData)

tumorNum=nrow(tumorData)

Type=c(rep("Normal",normalNum), rep("Tumor",tumorNum))

names(Type)=row.names(data)

Type=as.data.frame(Type)

pdf(file="heatmap.pdf", width=8, height=5)

pheatmap(t(data),

annotation=Type,

color=colorRampPalette(c(rep("blue",3), "white", rep("red",3)))(50),

cluster_cols=F,

show_colnames=F,

scale="row",

fontsize = 6,

fontsize_row=6,

fontsize_col=6)

dev.off()

data=t(tumorData)

col=rainbow(nrow(data), s=0.7, v=0.7)

pdf(file="barplot.pdf", width=20, height=10)

par(las=1,mar=c(6,5,2,15),mgp=c(3,0.1,0),cex.axis=1.5)

a1 = barplot(data,col=col,yaxt="n",ylab="Relative Percent",xaxt="n",cex.lab=1.8)

a2=axis(2,tick=F,labels=F)

axis(2,a2,paste0(a2*100,"%"))

axis(1,a1,labels=F)

par(srt=60,xpd=T);text(a1,-0.02,colnames(data),adj=1,cex=0.35);par(srt=0)

ytick2 = cumsum(data[,ncol(data)])

ytick1 = c(0,ytick2[-length(ytick2)])

legend(par('usr')[2]*0.98,par('usr')[4],legend=rownames(data),col=col,pch=15,bty="n",cex=1.2)

dev.off()

pdf(file="corHeatmap.pdf", width=11, height=11)

par(oma=c(0.5,1,1,1.2))

tumorData=tumorData[,colMeans(tumorData)>0]

M=cor(tumorData)

corrplot(M,

order="hclust",

method = "color",

addCoef.col = "black",

diag = TRUE,

tl.col="black",

number.cex=0.75,

col=colorRampPalette(c("blue", "white", "red"))(50))

dev.off()

install.packages("colorspace")

install.packages("stringi")

install.packages("ggplot2")

if (!requireNamespace("BiocManager", quietly = TRUE))

install.packages("BiocManager")

BiocManager::install("org.Hs.eg.db")

BiocManager::install("DOSE")

BiocManager::install("clusterProfiler")

BiocManager::install("enrichplot")

library("clusterProfiler")

library("org.Hs.eg.db")

library("enrichplot")

library("ggplot2")

pvalueFilter=0.05

qvalueFilter=0.05

colorSel="qvalue"

if(qvalueFilter>0.05){

colorSel="pvalue"

}

setwd("C:\\biowolf\\Diagnostic\\08.GO")

rt=read.table("diff.txt", header=T, sep="\t", check.names=F)

genes=as.vector(rt[,1])

entrezIDs=mget(genes, org.Hs.egSYMBOL2EG, ifnotfound=NA)

entrezIDs=as.character(entrezIDs)

gene=entrezIDs[entrezIDs!="NA"]

kk=enrichGO(gene=gene, OrgDb=org.Hs.eg.db, pvalueCutoff=1, qvalueCutoff=1, ont="all", readable=T)

GO=as.data.frame(kk)

GO=GO[(GO$pvalue<pvalueFilter & GO$qvalue<qvalueFilter),]

write.table(GO, file="GO.txt", sep="\t", quote=F, row.names = F)

showNum=10

if(nrow(GO)<30){

showNum=nrow(GO)

}

pdf(file="barplot.pdf", width=10, height=7)

bar=barplot(kk, drop=TRUE, showCategory=showNum, split="ONTOLOGY", color=colorSel) + facet_grid(ONTOLOGY~., scale='free')

print(bar)

dev.off()

pdf(file="bubble.pdf", width=10, height=7)

bub=dotplot(kk, showCategory=showNum, orderBy="GeneRatio", split="ONTOLOGY", color=colorSel) + facet_grid(ONTOLOGY~., scale='free')

print(bub)

dev.off()

install.packages("colorspace")

install.packages("stringi")

install.packages("ggplot2")

if (!requireNamespace("BiocManager", quietly = TRUE))

install.packages("BiocManager")

BiocManager::install("org.Hs.eg.db")

BiocManager::install("DOSE")

BiocManager::install("clusterProfiler")

BiocManager::install("enrichplot")

library("clusterProfiler")

library("org.Hs.eg.db")

library("enrichplot")

library("ggplot2")

pvalueFilter=0.05

qvalueFilter=0.05

colorSel="qvalue"

if(qvalueFilter>0.05){

colorSel="pvalue"

}

setwd("C:\\biowolf\\Diagnostic\\09.KEGG")

rt=read.table("diff.txt", header=T, sep="\t", check.names=F)

genes=as.vector(rt[,1])

entrezIDs=mget(genes, org.Hs.egSYMBOL2EG, ifnotfound=NA)

entrezIDs=as.character(entrezIDs)

rt=cbind(rt,entrezID=entrezIDs)

gene=entrezIDs[entrezIDs!="NA"]

kk=enrichKEGG(gene=gene, organism="hsa", pvalueCutoff=1, qvalueCutoff=1)

KEGG=as.data.frame(kk)

KEGG$geneID=as.character(sapply(KEGG$geneID,function(x)paste(rt$id[match(strsplit(x,"/")[[1]],as.character(rt$entrezID))],collapse="/")))

KEGG=KEGG[(KEGG$pvalue<pvalueFilter & KEGG$qvalue<qvalueFilter),]

write.table(KEGG, file="KEGG.txt", sep="\t", quote=F, row.names = F)

showNum=30

if(nrow(KEGG)<showNum){

showNum=nrow(KEGG)

}

pdf(file="barplot.pdf", width=8, height=7)

barplot(kk, drop=TRUE, showCategory=showNum, color=colorSel)

dev.off()

pdf(file="bubble.pdf", width = 8, height = 7)

dotplot(kk, showCategory=showNum, orderBy="GeneRatio", color=colorSel)

dev.off()
